# Mechanistic Insights into the Selective Synthesis of 4*H*-Pyran Derivatives On-Water Using Naturally Occurring Alginate from *Sargassum muticum:* Experimental and DFT Study

**DOI:** 10.3390/gels8110713

**Published:** 2022-11-04

**Authors:** Khaoula Oudghiri, Zahira Belattmania, Hamid Elmouli, Salaheddine Guesmi, Fouad Bentiss, Brahim Sabour, Lahoucine Bahsis, Moha Taourirte

**Affiliations:** 1 Laboratoire de Recherche en Développement Durable et Santé, Faculté des Sciences et Techniques de Marrakech, Université Cadi Ayyad, Marrakech 40000, Morocco; 2 Laboratory of Plant Biotechnology, Ecology and Ecosystem Valorization—URL CNRST N=10, Faculty of Sciences El Jadida, University Chouaib Doukkali, P.O. Box 20, El Jadida 24000, Morocco; 3 Laboratoire de Chimie de Coordination et d’Analytique (LCCA), Département de Chimie, Faculté des Sciences, Université Chouaïb Doukkali, P.O. Box 20, El Jadida 24000, Morocco; 4 Laboratory of Catalysis and Corrosion of Materials, Faculty of Sciences, University Chouaib Doukkali, P.O. Box 20, El Jadida 24000, Morocco; 5 Materials and Transformations Unit, University of Lille, CNRS, INRAE, Centrale Lille, UMR 8207-UMET, F-59000 Lille, France; 6 Laboratoire de Chimie Analytique et Moléculaire, LCAM, Faculté Polydisciplinaire de Safi, Université Cadi Ayyad, Safi 46030, Morocco

**Keywords:** alginate, 4*H*-pyran, biopolymers, DFT calculations, NCI

## Abstract

The naturally occurring sodium alginate (SA) biopolymer from the *Sargassum muticum* (Yendo) Fensholt was employed as a green organocatalyst for the synthesis of 4*H*-pyran derivatives. The naturally extracted macromolecule was fully characterized using different analyses, including nuclear magnetic resonance (NMR), Fourier-transform infrared spectroscopy (FTIR), scanning electron microscopy (SEM), and Energy Dispersive X-ray Analysis (EDX). The catalytic activity of SA was investigated in the one-pot reaction between aldehydes, malononitrile, and 1,3-dicarbonyl compounds in water at room temperature, and the corresponding 2-amino-3-cyano-4*H*-pyran derivatives were obtained with good to excellent yields. This organocatalyst was easily separated from the reaction mixture and reused for at least two consecutive cycles without a significant loss of its catalytic activity or selectivity. From the mechanistic point of view, density functional theory (DFT) and NCI analyses were performed for the first time to explain the regioselectivity outcomes for the synthesis of 2-amino-3-cyano-4*H*-pyran derivatives using SA as a green organocatalyst.

## 1. Introduction

Sustainable chemistry has had great interest in the last decade, leading to the investigation and applications of natural polymers such as cellulose, alginate, and hemicellulose in many industrial areas [1,2,3,4]. Alginates, which are obtained from brown seaweed, play a vital role in the binding and mechanical properties of new materials [5,6]. Its mechanical properties depend on the average molecular weight and the distribution of the three types of molecular fragments (β-D-mannuronic (FM), α-L-guluronic (FG) acids, and heteropolymeric fractions (FMG)) [7,8]. These mechanical and biodegradability properties make this polysaccharide an excellent candidate in medical industries, textiles, the food industry, anticoagulant materials, and dentistry [8,9,10,11,12,13,14]. Alginates are also used in electrochemical sensors as coating agents of electrodes due to their low cost, easy availability, and non-toxic environmentally-friendly nature [15,16]. Several brown algal species can be a source of the alginate biopolymer such as *Laminaria hyperborea*, *Durvillaea antarctica*, *Ascophyllum nodosum*, *Ecklonia maxima*, *Saccharina latissima*, *Lessonia nigrescens*, *Macrocystis pyrifera*, and *Lessonia trabeculata* [17,18]. Moreover, the presence of functional groups in the sodium alginate structure makes it an excellent polymeric skeleton for the coordination of transition metal ions [6,19]. In addition, this biopolymer is defined by its high surface area and chemical modifying capacity, making it an interesting biodegradable polymer for many catalytic applications [20,21].

The pyran derivatives are reported as important containing molecules in many natural products [22]. These compounds are characterized by the presence of hetero-atoms, leading them to be used as important compounds in various applications such as biological, corrosion, and drug research [23,24,25]. Among pyran derivatives, 4*H*-pyran derivatives have great attention due to their important properties [26,27,28]. The development of an efficient synthetic method for the synthesis of 4*H*-pyran derivatives using greener conditions has garnered researchers attention over the last decade [29]. We report here the extraction and purification of a naturally-occurring sodium alginate biopolymer from the *Sargassum muticum* (Yendo) Fensholt. The obtained biopolymer exhibits a high catalytic activity and selectivity as an organocatalyst for the synthesis of 2-amino-3-cyano-4*H*-pyran derivatives in water at room temperature, and their recovery/reuse has been also evaluated. Additionally, mechanistic studies were performed using Density Functional Theory (DFT) calculations and non-covalent interaction (NCI) analysis to account for the selectivity of this organocatalyst for the synthesis of 4*H*-pyran derivatives.

## 2. Results and Discussion

### 2.1. Sodium Alginate (SA) Biopolymer Characterization

**Scheme 1:**
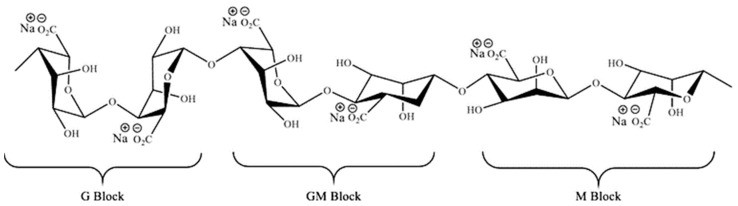
Molecular structure of sodium alginate (SA).

The structural analysis of the sodium alginate (SA) shows that is formed by a linear copolymer of two homopolymeric blocks of (1→4)-linked α-L-guluronic acid (G) and β-D-mannuronic acid (M) monomers and heteropolymorphic fractions GM, see Scheme 1 [30,31]. All these monomers contain a carboxylate and two hydroxyl groups which can act as a bifunctional heterogeneous organocatalyst for the electrophilic and nucleophilic reactions assisted by carboxylate groups and/or hydrogen bonding [32,33,34,35]. To get more light on the surface and chemical structural properties, the obtained natural-occurring sodium alginate (SA) was examined by SEM, EDX, FTIR, and NMR analyses. A regular shape homogeneous with some wrinkles and pores with a relatively smooth surface of this biopolymer was observed (Figure S1). The heterogeneous and the presence of functional groups on the surface structure of the obtained SA could favor the adsorption process of small molecules via electrostatic, hydrogen bonding, and/or van der Waals interactions [36]. The purity of this natural-occurring biopolymer was firstly investigated by EDX analysis, and the results indicate the presence of oxygen, carbon, and sodium atoms. These findings confirm the purity of this naturally extracted alginate (Figure S1).

**Figure 1:**
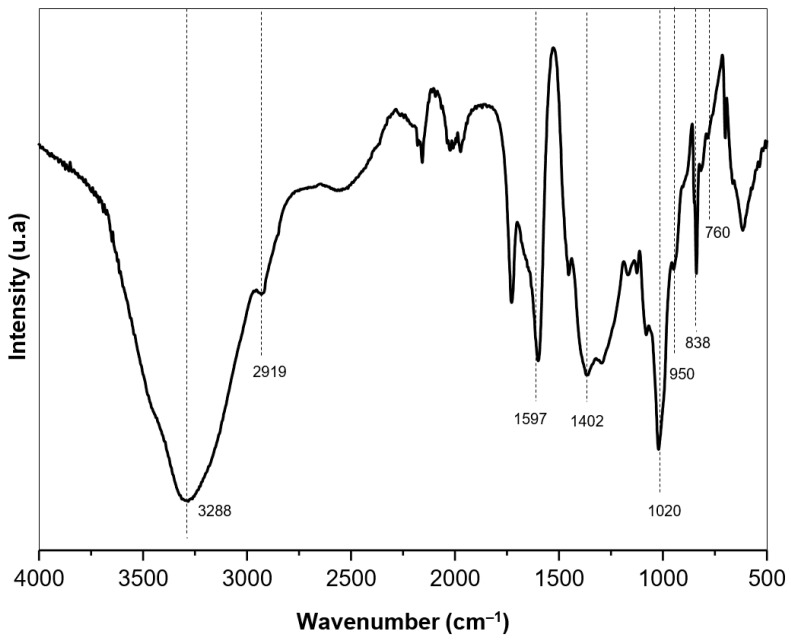
FTIR spectrum of SA from *Sargassum muticum*.

The FTIR spectrum of SA obtained from *Sargassum muticum* is illustrated in Figure 1. The peak that appeared at 3288 cm^−1^ is assigned to the stretching vibration of hydroxyl groups (OH). The observed peak at 2919 cm^−1^ is attributed to asymmetric stretching vibrations of the carboxylate group(O=C–O). The characteristic peak of alginate is observed at 1597 cm^−1^, indicating the asymmetric stretching vibrations of carboxylate salt ions. The peak located at 1402 cm^−1^ could be attributed to the deformation vibration of the (C–OH) group and the symmetric stretching vibration of the carboxylate group. It is also reported that, when the proton of the carboxylic acid group was displaced by a sodium atom (monovalent ion), the characteristic peaks of carboxylate salt ions appeared around 1600 and 1400 cm^−1^, respectively [36]. The characteristic band of the pyranose ring is observed at 1020 cm^−1^ assigned to C–O, and C–C stretching vibrations. Two other characteristic bands of alginate are located in the 1000 to 800 cm^−1^ region. The first one is found at 950 cm^−1^ which corresponds to the C–H deformation vibration of β-D-mannuronic acid, while the second peak is observed at 838 cm^−1^ which can be attributed to mannuronic acid [18,37].

**Scheme 2:**
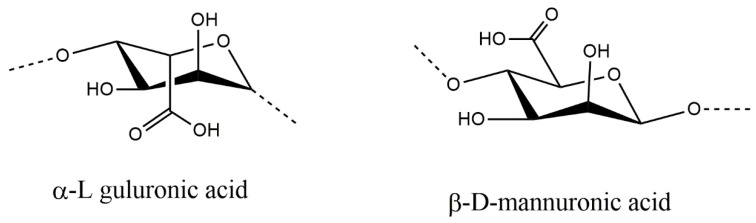
α-L guluronic acid andβ-D-mannuronic acid structures.

**Figure 2:**
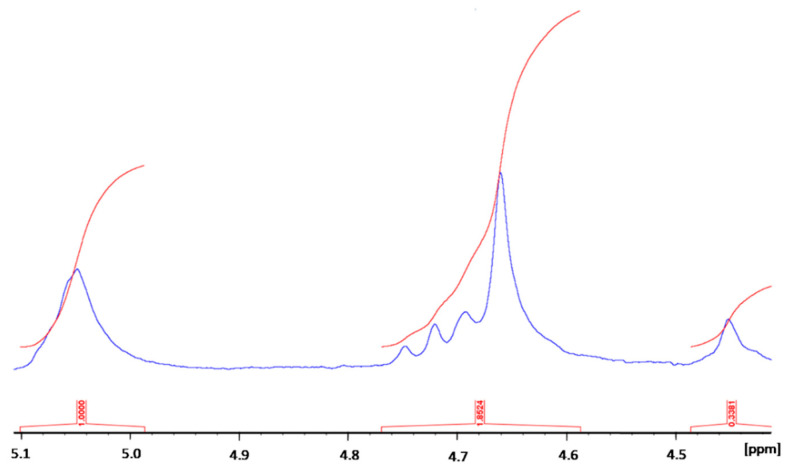
^1^H NMR spectrum of Na-alginate (SA) from *Sargassum muticum*.

The ^1^H NMR spectrum of the sodium alginate extracted from *S. muticum* (Figure 2) showed three characteristic signals: the first signal recorded at 5.04 ppm is related to the anomeric hydrogen of guluronic acid (Scheme 2), the second signal at 4.66–4.74 ppm corresponds to the anomeric hydrogens of mannuronic acid (M1) and the H-5 of the alternating blocks (GM-5). The third signal detected at 4.45 ppm is linked to the H-5 of the guluronic acid residues in the homopolymeric G regions. Following the equations of Grasdalen et al. (1979) [38], the guluronic acid and mannuronic acid fractions (F_G_, F_M_) as well as the dyads fractions (F_GG_, F_MM_, F_MG_/_GM_) were calculated based on the area of the aforementioned signals (Figure 2). The M/G ratio of SA from *S. muticum* was about 1.19, representing a relatively high proportion of mannuronic acid (F_M_ = 54%) than the α-L guluronic acid (F_G_ = 46%). Furthermore, the homopolymorphic fractions of the extracted SA are markedly low (F_MM_ = 24%; F_GG_ = 15%) compared to the heteropolymeric fractions (F_MG_ + F_GM_ = 60%). These results indicated that SA from *S. muticum* provides highly elastic gels. According to Draget et al. (2006) [39], the alginates gel elasticity raises in the following order: GG blocks < MM blocks < MG blocks. The GG regions are not very abundant in SA from *S. muticum*, although they have a higher affinity for divalent ions, explaining its potential effect as an organocatalyst. 

All of these findings confirm the chemical structure and functional groups of the naturally occurring sodium alginate obtained from *Sargassum muticum*. The presence of a large number of carboxyl groups and hydroxyl groups makes this organocatalyst an excellent candidate for electrophilic and nucleophilic reactions. In addition, this SA biopolymer can accelerate chemical reactions via establishing hydrogen bonding with starting materials and intermediate complex or reaction activation through reactants deprotonation [40].

### 2.2. Catalytic Activity

**Scheme 3:**
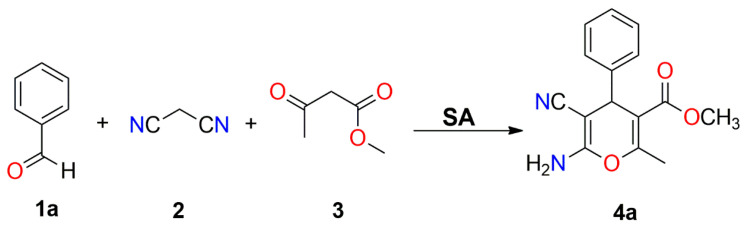
The synthesis of 2-amino-3-cyano-4*H*-pyran (4a) is catalyzed by naturally occurring SA.

To evaluate the catalytic activity of the obtained macromolecule, the SA was employed for the synthesis of 2-amino-3-cyano-4*H*-pyran derivatives under greener conditions. As the starting point of our exploration, the one-pot three-component reaction of benzyl aldehyde (1a), malononitrile (2), and methyl 3-oxobutanoate (3) were chosen as model reactions. Different reaction conditions such as alginate amount, solvent effect, reaction time, and temperature were also investigated (Scheme 3, Table 1).

The controlled experiments have shown that this reaction does not take place in the absence of the SA biopolymer (entry 1). The solvent effect using only 40 mg of this biopolymer was firstly investigated (entries 2–6). The results show that the water solvent conducts an excellent yield of 4a product after 24 h at room temperature. The effect of different solvents was also investigated. The findings confirm that water was the most effective solvent, while the use of other solvents such as acetone and ethanol resulted in lower yields. These preliminary results led to the investigation of the amount effect of the SA on the yield of this reaction. Interestingly, the polymer loading could be further increased to 100 mg, leading to an excellent yield of the 2-amino-3-cyano-4*H*-pyran (93.7%) within 10 min in water at room temperature (entry 10, Table 1). The solvent effect under similar conditions was investigated using hexane as a solvent. The corresponding product was obtained in moderate yield after 2 h (entry 11, Table 1). The temperature effect was also investigated using water as a solvent (entries 12–14), and the results indicate that the increase of the reaction temperature conducts only to good yields of the corresponding 4*H*-pyran (4a). It was concluded that the optimal conditions involved 100 mg of the SA in water at room temperature (entry 10, Table 1). These greener conditions make this naturally extracted macromolecule a sustainable catalyst for the selective synthesis of 2-amino-3-cyano-4*H*-pyran derivatives.

**Figure 3:**
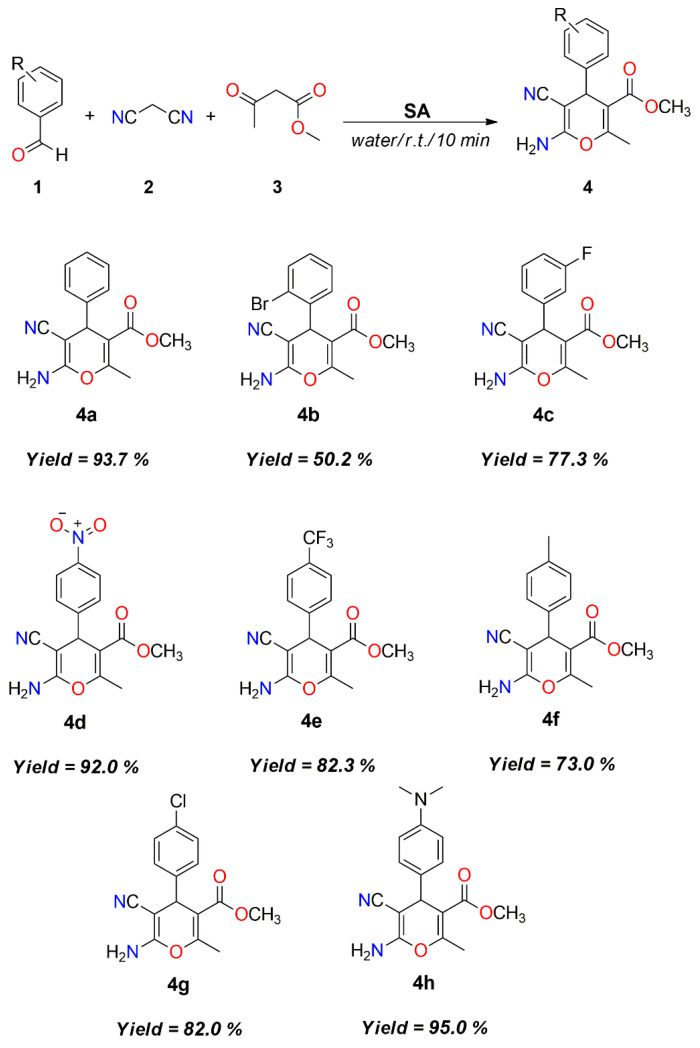
Synthesis of some 4*H*-pyran derivatives (**4a**–**f**) using the naturally occurring SA.

From a mechanistic point of view, the reaction rate of the organocatalytic reaction depends on the electron-donating/accepting nature of the substituents and their position on reactants [41,42]. After the optimum conditions are obtained, the versatility of the naturally extracted biopolymer was then explored structurally some selected aldehydes, and the results are summarized in Figure 3. The obtained results of the selective synthesis of 2-amino-3-cyano-4*H*-pyran derivatives using SA as an organocatalyst show that the aldehydes with electron-withdrawing or electron-donating groups required a shorter reaction time, which is caused by the nucleophilic attack process of the corresponding anion of malononitrile to the carbon atom of aldehyde function (products 4a and 4e–h). Consequently, the corresponding *Knoevenagel* reaction and *Michael* addition will be progressed more readily. Unfortunately, compound 4d was obtained with a moderate yield. The position of electron-withdrawing or donating groups on aromatic aldehyde has a significant influence on the rate of this catalytic reaction. Both ortho- and meta-position reduce the rate of this catalytic reaction due to the formation of hydrogen bonds between reactants through the reaction process, which leads to the formation of more stable reaction intermediates [43,44]. These findings were confirmed by the lower yield of compounds 4b and 4c, see Figure 3. Moreover, all corresponding 4*H*-pyran derivatives were obtained in good to excellent yields and did not require any further purification by conventional methods, which is a fundamental interest in the development of new ‘‘green’’ processes that can selectively yield the 2-amino-3-cyano-4*H*-pyran derivatives.

### 2.3. Mechanistic Studies

**Scheme 4:**
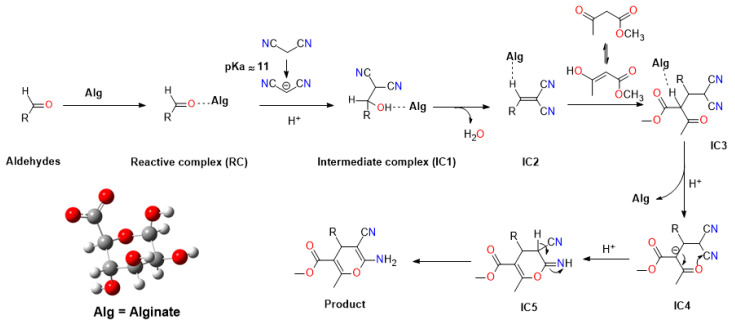
Generally accepted mechanism for the synthesis of 4a catalyzed by SA.

In order to explain these experimental findings and to get more light on the selective synthesis of 4*H*-pyran derivatives using sodium alginate (SA), density functional theory (DFT) calculations were conducted using the B3LYP/6-31G (d, p) method by choosing an appropriate model for SA, as is illustrated in Scheme 4 with water as a solvent. These DFT calculations were investigated for the first time on the accepted mechanism for the selective synthesis of 4*H*-pyran derivatives using condensation reaction of benzaldehyde (1), malononitrile (2), and methyl 3-oxobutanoate (3) to give the corresponding 2-amino-3-cyano-4*H*-pyran derivative (4a) (Scheme 3).

**Figure 4:**
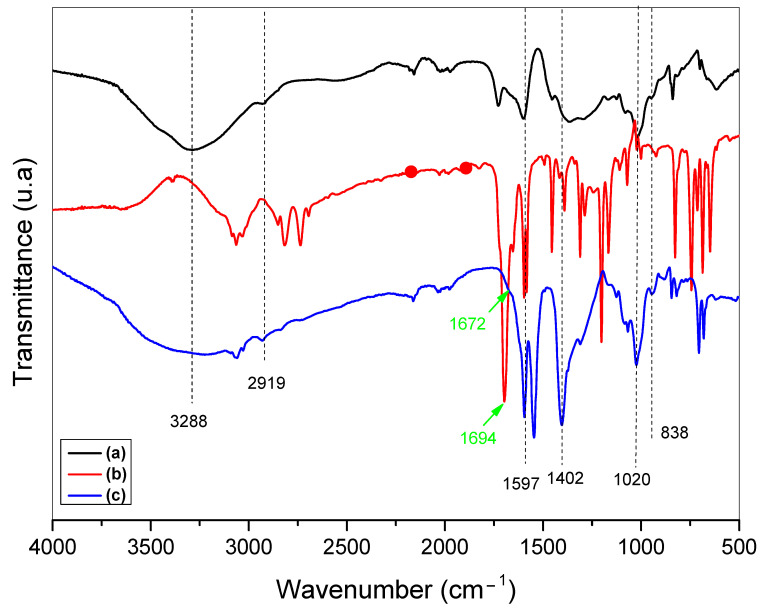
FTIR spectra (**a**) SA from *Sargassum muticum*, (**b**) benzaldehyde, and (**c**) SA after the adsorption of benzaldehyde.

The first step is the formation of a reactive complex (RC) between aldehyde derivatives and alginate biopolymer through hydrogen bonding. To demonstrate the formation of the RC, benzaldehyde was reacted with SA at room temperature, and then an FTIR spectrum was performed. The analysis of the characteristic bond of the benzaldehyde, C=O functional group, indicates a decrease in the value of this bond from 1694 to 1672 cm^−1^, confirming the modification in double bond nature, and an activated carbonyl was formed (Figure 4 and Figure S10) [45,46,47]. Moreover, a non-covalent analysis (NCI) was performed on RC intermediate to identify the interactive nature between benzaldehyde and SA (see Figure S11 in SI). The results confirm that vdW interactions and hydrogen bonds were located in RC between benzaldehyde and SA. The effect of the amount of SA on the pH of the reaction medium was also investigated in the first step. The results indicate the increase in the amount of SA result in a basic aqueous solution (see Table S1). The obtained results show that for 100 mg of SA, pH = 10.34 which is almost close to the pka of malononitrile, pKa ≈ 11, and consequently the easy deprotonation of malononitrile leads to the formation of cyanocinnamonitriles, which reacted with formed RC (Scheme 3). These findings are in good agreement with the reported results of Jonnalagadda et al. using NaOH as a catalyst in water [48]. The next step is the formation of an intermediate complex (IC1) followed by the elimination of a water molecule (IC2). The presence of the enol form of 1,3-dicarbonyl components conducts the *Michael* addition reaction (IC3). The cyclization of the obtained IC4 and final tautomerization of the intermediate (IC5) results in the desired product (4a).

**Figure 5:**
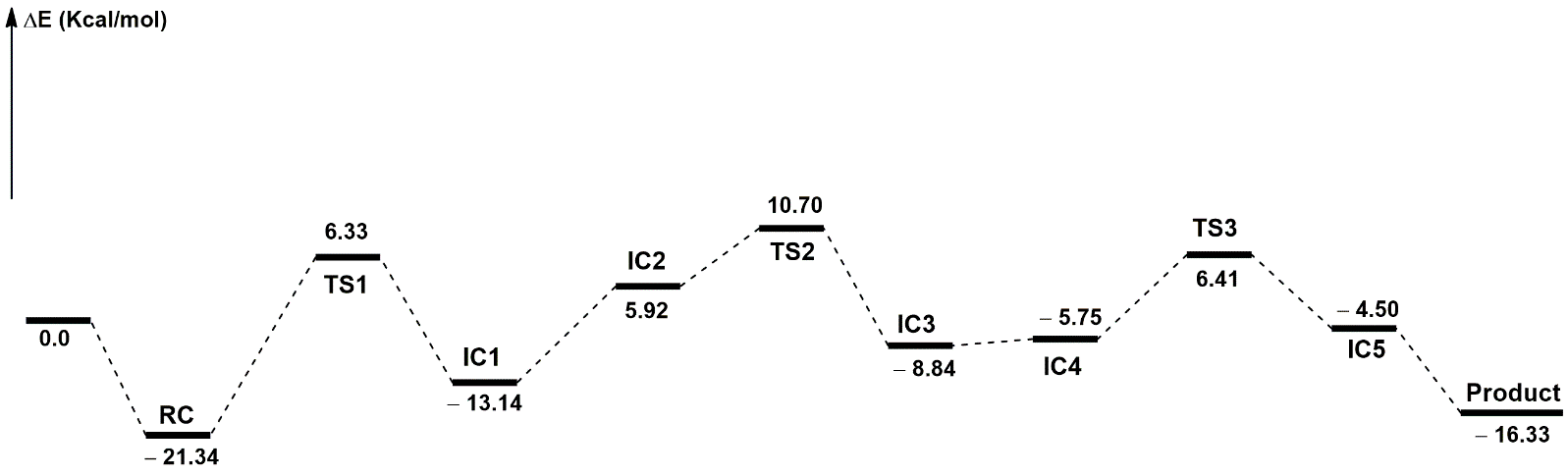
DFT-computed energy profile for the synthesis of 4a using SA as a catalyst in water. All values are reported in kcal/mol.

**Figure 6:**
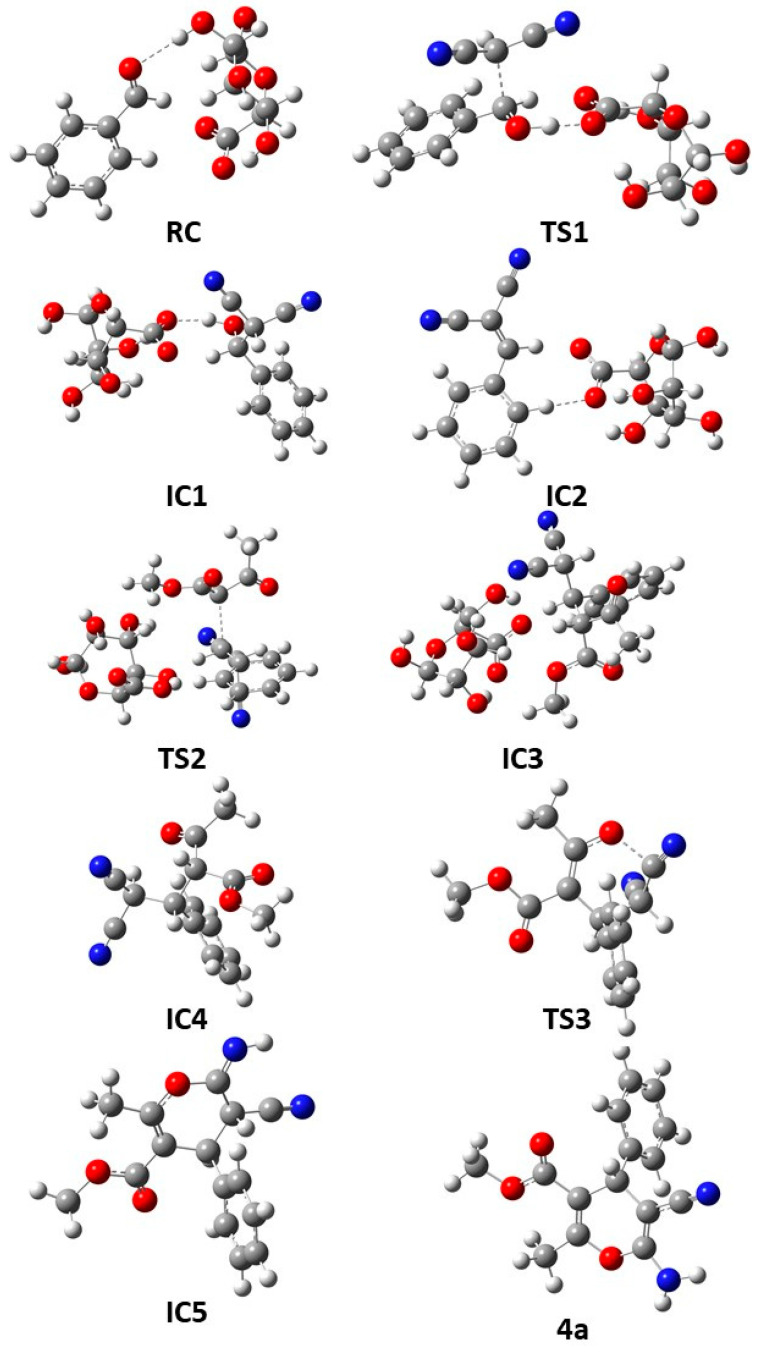
Optimized structures of all intermediates for the synthesis of 4a catalyzed by SA. For color codes: grey (carbon atoms), red (oxygen atoms), blue (nitrogen atoms) and white (hydrogen atoms).

To shed more light on the experimental results, DFT calculations were carried out using a model for the prepared alginate (see Scheme 3). In the first step, the optimized structure of the reactive complex (RC) was performed (Figure 5), and is illustrated in Figure 6. The formation of this RC is characterized by negative energy, indicating that the coordination reaction between benzaldehyde and alginate is exothermic by 21.34 kcal/mol (Figure 5). This high stability of RC is due to the hydrogen bond between benzaldehyde and alginate structure, as confirmed by NCI analysis (Figure S11 in SI). This hydrogen bond is located between the carbonyl group of aldehyde and the hydroxyl groups on the surface of alginate, which can facilitate the activation of nucleophile attacks on the carbon atom of the aldehyde function [49,50,51,52]. The reaction between the RC and cyanocinnamonitrile conducts the formation of an intermediate complex (IC1). This step is exothermic by 13.14 kcal/mol. The formation of the IC1 is performed through a transition state (TS1). The result indicates that this TS is endothermic by 6.33 kcal/mol, and its optimized structure is illustrated in Figure 6. Interestingly, this activation energy was found to be lower than associated with the non-catalyzed reaction (NTS = 39.16 kcal/mol) (Figure S12 in SI), which led us to confirm the catalytic effect of the SA as an organocatalyst for the synthesis of 2-benzylidene malononitrile in water at room temperature. These results are in good agreement with the experimental observations reported by Kolvari et al. [40], indicating that biomass can be an excellent candidate for the synthesis of benzylidene malononitrile derivatives. Subsequently, IC2 was formed by removing water molecules, with an energy barrier equal to 19.06 kcal/mol. The step is also endothermic in the non-catalytic reaction (NIC1 = 5.30 kcal/mol). Michael’s addition of the intermediate complex IC2 with methyl 3-oxobutanoate (3) using SA as the catalyst proceeds via the transition state TS2 and results in the formation of the intermediate IC3. This TS is characterized by an endothermic barrier (*ca* 10.70 kcal/mol), and its structure is illustrated in Figure 6. This transition state is more stabilized than that of the first step, namely TS1, which can be explained by the presence of more hydrogen bonds between this TS and the alginate structure (Figure 5 and Figure 6). In the non-catalytic reaction, this TS is characterized by high activation energy, which is 35.23 kcal/mol (Figure S12 in SI). Interestingly, the removal of alginate moieties and a proton atom from IC3 leads to the formation of IC4, which is exothermic by 5.75 kcal/mol (see Figure 6). These findings seem to be mediated by the delocalization of the π electrons from the formed carbanion and C=O group, which enhanced the nucleophilic attack carbon atom of the cyanuric group (CN) by the oxygen atom, leading to the formation of a six-ring heterocycle via TS3 (Scheme 4). The cyclization of the obtained IC4 led to the formation of IC5 through TS3, which is endothermic by 6.41 kcal/mol, which is lower compared to the obtained TS in the non-catalytic reaction, NTS3 = 41.08 kcal/mol. The desired product (4a) was obtained by the tautomerization of the intermediate (IC5). This step is exothermic by 16.33 kcal/mol for a catalytic reaction and 21.64 kcal/mol for a non-catalytic reaction (Figure 6 and Figure S12). Hence, the amphoteric character of water molecules has an important role in the synthesis of the 4*H*-pyrans compared to organic solvents, confirmed by the high reaction rate in water compared to non-polar solvents such as hexane (see Table 1) [48]. These results show that all energetic barriers are lower, allowing us to explain that this catalytic reaction for the synthesis of 4a can be carried out in the water at room temperature, which is in excellent agreement with the experimental observations.

**Figure 7:**
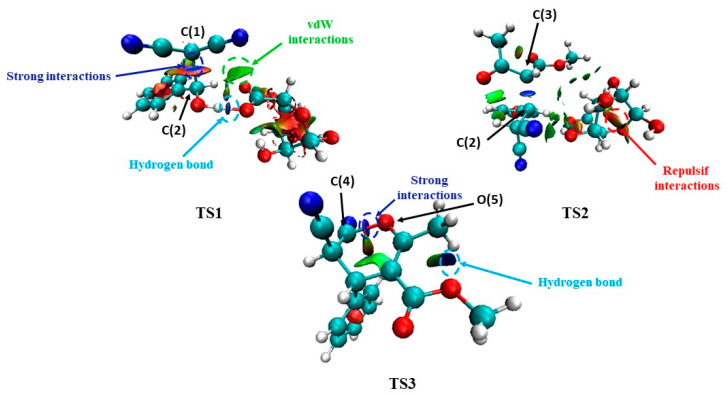
NCI analysis of TSs for the one-pot reaction for the synthesis of 4a catalyzed by SA (s = 0.3). For color codes: green (carbon atoms), red (oxygen atoms), blue (nitrogen atoms) and white (hydrogen atoms).

NCI analysis at TSs was performed to interpret the origin of bonding interaction in the catalytic reaction of the 2-amino-3-cyano-4*H*-pyran (4a) using SA as an organocatalyst. As can be seen in Figure 7, although some weak interactions between the reagents and SA appear blue, indicating strong interactions or hydrogen bonds, the surface colored in green suggests the existence of a van der Waals (vdW) attraction. The small yellowish and red regions suggest the presence of a weak to strong repulsion. In the TS1 case, the results of NCI analysis indicate the presence of a strong interaction between C1 and C2, which conducts to the formation of the corresponding intermediates complex (IC1). The weak to strong repulsion interaction (yellowish to red surface color) located in a TS2 explain its high-energy barrier compared with TS1. Finally, the NCI analysis at TS3 shows a strong interaction between C4 and O5, indicating the favorable interaction preceding the C4-O5 single bond formation. These excellent agreements between the experimental results and the DFT calculations give a substantial basis to shed more light on the selective synthesis of the corresponding 2-amino-3-cyano-4*H*-pyran derivatives using sodium alginate as an organocatalyst in water.

### 2.4. Reusability Test

A reusability test of the naturally occurring SA for the synthesis of 4*H*-pyran was performed. The reactions of benzyl aldehyde (1a), malononitrile (2), and methyl 3-oxobutanoate (3) in the presence of SA in water at room temperature were chosen as model reactions. The results indicate that after two cycles, the SA organocatalyst gives an excellent yield (80%). Importantly, this organocatalyst shows that the catalytic activity and selectivity did not significantly decrease. The recovered biopolymer alginate was analyzed by SEM, EDX, and FTIR spectroscopies. The SEM analysis shows that the surface of this recovered material is very smooth and not porous (Figure S1). The EDX analysis indicates that an almost identical atomic percentage was obtained in both cases before and after the catalyst. Moreover, the EDX mapping was also performed for the fresh and recycled SA to get more evidence on the distribution of its characteristic elements such as sodium, oxygen, and carbon atoms (Figure S1). The results show that the EDX mapping images give a different color mapping compared to the fresh SA due to the low concertation of sodium atoms after the catalytic reaction. The functional groups on the recovered SA biopolymer were investigated by FTIR analysis (see Figure S13 in SI). The spectroscopy analyses of the recovered SA are almost similar to the fresh material, which could explain the slight loss of its catalytic activity after the first cycle and confirms the stability of this organocatalyst after two cycles. The high catalytic activity, biodegradability, simple separation, and recyclability of sodium alginate make it more environmentally benign.

### 2.5. Comparison of the Catalytic Activity of Our Organocatalyst with Other Reported Protocols

To show the merits of the SA as an organocatalyst for the selective synthesis of the 4*H*-pyran derivatives, its catalytic performance was compared with other reported catalytic protocols (see Table 2). The reaction benzyl aldehyde (1a), malononitrile (2), and methyl 3-oxobutanoate (3) was chosen as a model substrate. The results of this comparison show that SA is similar to the catalytic protocol that used dibutylamine, a strong base, in terms of reactivity and reaction time. Similar results to the KF-Al_2_O_3_ and Ta-MOF nanostructured catalysts were reported in terms of reactivity with a long reaction time. Both catalysts, BaFe_12_O_19_@ IM and DES (Urea-ChCl) need a high reaction temperature (~ 80 °C) and more reaction time. Remarkably, in our catalytic system using sodium alginate (SA) as an organocatalyst, the addition of a strong base or the increase of reaction temperature to activate the reaction is not needed and the SA catalyst is easily separated from the reaction mixture by simple filtration. All of the above-mentioned qualities led us to confirm that SA is an excellent bio-functional organocatalyst for the selective synthesis of 2-amino-3-cyano-4*H*-pyran derivatives.

## 3. Conclusions

To summarize, we reported the preparation of a naturally occurring alginate from the invasive brown seaweed *Sargassum muticum* and its application as a greener catalyst for the one-pot three-component reaction for the synthesis of 2-amino-3-cyano-4*H*-pyran derivatives in water at room temperature, which takes on all green chemistry conditions. Mild conditions, the absence of transition metal, short reaction time, reusability, and high to excellent yields of products make this protocol a great way for the synthesis of functionalized and medicinal 4*H*-pyrans derivatives. Moreover, for the first time, the mechanism for the synthesis of 2-amino-3-cyano-4*H*-pyran derivatives was investigated through DFT calculations and NCI analysis. The obtained results are in excellent agreement with the experimental findings which can allow us to understand the catalytic activity of SA as a bio-functional organocatalyst for the selective synthesis of 2-amino-3-cyano-4*H*-pyran derivatives. We anticipate that these facts will help chemists to investigate other biopolymers for the selective synthesis of 4*H*-pyrans and/or other organic compounds.

## 4. Materials and Methods

### 4.1. Materials

All reagents were purchased from Merck (Merck Group, Darmstadt, Germany), Fluka (Chemie GmbH, Buchs, Switzerland) and Sigma-Aldrich (Chemie GmbH, Riedstr, Taufkirchen, Germany). The Merck 0.2 mm silica gel 60 F-254Al-plates were used for the analytical thin layer chromatography (TLC) for monitoring reactions.

### 4.2. Preparation of the Naturally-Occurring Sodium Alginate

Sodium alginate was extracted from the invasive species *Sargassum muticum* collected from the Atlantic coast of Morocco. A solution of 2% formalin was used to hydrate the obtained dried biomass of *S. muticum* for 24 h and then washed with distilled water. The next step is soaking this biomass in 0.2 N HCl for 24 h, then washing it with distilled water for a second time and extracting it with 2% Na_2_CO_3_, filtering it, and centrifuging (4500× *g*, 20 min). The supernatant was precipitated with 95% ethanol, conducting to the desired biopolymer which is dried at 50 °C until constant weight.

### 4.3. Characterization

The TESCAN-VEGA3 SEM (Scientific and technical instruments, Brno, Czech Republic) was performed to investigate the surface morphology of the prepared naturally-occurring alginate biopolymer using an accelerating voltage of 20 kV. To identify the specific elemental composition and mapping on the surface of the obtained biopolymer, an energy dispersive X-ray (EDAX) analysis was employed. The functional groups of naturally-occurring sodium alginate were conducted using FTIR (Bruker VERTEX 70, Bruker Corporation, Hamburg, Germany) analysis. The transparent pellets for FTIR analyses were prepared by mixing the obtained solid state of sodium alginate with dried solid of KBr and were operated at 4 cm^−1^ over a range of 4000–400 cm^−1^.

An AV II 400 MHz, 9.4T spectrometer (Proton Larmor frequency of 400.33 MHz, Bruker Corporation, Billerica, MA, USA) was used for ^1^H NMR analysis of naturally-occurring sodium alginate. A 5 mm Triple Resonance Broadband Inverse (TBI) probe (Bruker Corporation, Billerica, MA, USA) was applied at 343 K with a 16 K size of free induction decay covering a sweep width of 4800 Hz. During the relaxation delay and mixing time, a presaturation was applied. A 0.5 for line broadening before Fourier transformation in one dimension was used in the exponential multiplication apodization functions with 32 transients as a number of scans.

### 4.4. General Experimental Procedure for the Synthesis of 2-Amino-3-Cyano-4H-Pyran Derivatives

Benzaldehyde derivatives, methylacetoacetate, and malononitrile (molar ratio 2:2:2) were dissolved in 5 mL of distilled water in the presence of 100 mg alginate as a catalyst. The reaction mixture was stirred at room temperature for 10 min. After reaction completion, the crude product was dissolved in hot ethanol and the catalyst was recovered by simple filtration for the next reuse. The corresponding products were recrystallized in hot ethanol, then filtered and dried in an oven at 50 °C.

### 4.5. Computational Methods

The DFT calculations were performed with the Gaussian09 package (Revision C.01; Gaussian, Inc.: Wallingford, CT, USA) [58], and the geometry optimizations of all intermediates were performed using the B3LYP method with the 6-31G (d, p) as a basis set [59,60]. The water solvent was applied in all optimized structures using the CPCM model [61]. The frequency calculations were also carried out to identify each stationary point or transition state. The stationary points were characterized by the positive vibrational frequency and the transition states were identified by one imaginary frequency. Non-covalent interactions (NCI) analyses were done to the most stable conformation of catalytic reaction intermediates using Multiwfn [62] and their visualization was realized by VMD software (version 1996, USA) [63]. Color mapping was used to identify the nature of interactions such as hydrogen bonds, van der Waals interactions, and steric repulsions, and the corresponding colors are blue, green, and red, respectively [64].

## Figures and Tables

**Table 1:** Optimization of reaction conditions for the synthesis of **4a** catalyzed by naturally occurring SA ^a^.

Entry	Catalyst	Loading (mg)	Solvent	Time (h)	Temperature (°C)	Yield (%) ^b^
1	Neat	-	H_2_O	24	r.t.	0
2	SA	40	H_2_O	24	r.t.	97.3
3	SA	40	Ethanol	24	r.t.	57.3
4	SA	40	Acetonitrile	24	r.t.	80.3
5	SA	40	Hexane	24	r.t.	88.3
6	SA	40	Acetone	24	r.t.	39.3
7	SA	5	H_2_O	0.2	r.t.	16.8
8	SA	20	H_2_O	0.2	r.t.	35.8
9	SA	40	H_2_O	0.2	r.t.	73.2
10	SA	100	H_2_O	0.2	r.t.	93.7
11	SA	100	Hexane	2	r.t.	64.0
12	SA	40	H_2_O	3	40	56.4
13	SA	40	H_2_O	3	60	72.4
14	SA	40	H_2_O	3	80	83

^a^ Reaction Conditions: benzaldehyde (1 mmol), malononitrile (1 mmol), dimedone (1 mmol), water (5 mL), and catalyst at room temperature. ^b^ Isolated yields.

**Table 2:** Comparative results of catalytic efficiency for the selective synthesis of compound **4a**.

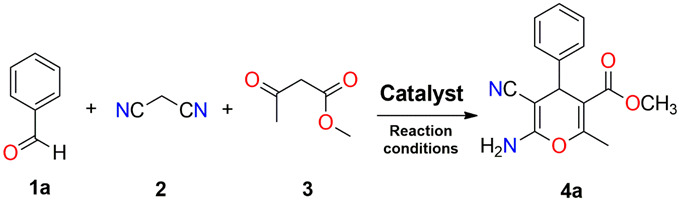					
Catalyst	Loading	Conditions	Time (h)	Yield (%)	Ref.
Dibutylamine	2.5 ^a^	Neat; r.t	0.14	98	[53]
BaFe_12_O_19_@ IM	3.12 ^b^	Refux; EtOH	0.25	96	[54]
DES (Urea-ChCl)	30 ^a^	EtOH; 80 °C	0.5	88	[55]
KF-Al_2_O_3_	5 ^a^	EtOH; r.t	5	90	[56]
Ta-MOF nanostructures	0.003 ^b^	EtOH; r.t	0.5	71	[57]
Sodium Alginate (SA)	0.100 ^b^	water; r.t	0.17	97.3	This work

^a^ Loading in mol%; ^b^ Loading in g. IM = Irish moss; Ta-MOF = Tantalum-Based Metal-Organic Framework. DES = Deep eutectic solvent; ChCl = Choline chloride.

## Data Availability

Not applicable.

## Supplementary Materials

The following supporting information can be downloaded at: https://www.mdpi.com/article/10.3390/gels8110713/s1, Figure S1: (A) SEM micrographs, (B) EDX and (C) EDX mapping analyses of fresh and recovered natural-occurring SA surface.; Figure S2: ^1^H NMR Spectrum of compound 4a; Figure S3: ^1^H NMR Spectrum of compound 4b; Figure S4. ^1^H NMR Spectrum of compound 4c; Figure S5. ^1^H NMR Spectrum of compound 4d; Figure S6. ^1^H NMR Spectrum of compound 4e; Figure S7. ^1^H NMR Spectrum of compound 4g; Figure S8. ^1^H NMR Spectrum of compound 4f; Figure S9. ^1^H NMR Spectrum of compound 4h; Figure S10. FTIR spectrum of benzaldehyde; Figure S11. NCI analysis of RC intermediate (s = 0.34); Figure S12. (A) Generally accepted mechanism and (B) DFT-computed energy profile for the non-catalytic reaction for the synthesis of 4a in water. All values are reported in kcal/mol; Figure S13. FTIR spectrum of the recovered SA after two cycles; Table S1. Effect of the amount of SA on the pH of aqueous solution

## References

[1] George A., Sanjay M.R., Srisuk R., Parameswaranpillai J., Siengchin S. (2020). A Comprehensive Review on Chemical Properties and Applications of Biopolymers and Their Composites. Int. J. Biol. Macromol..

[2] Kartik A., Akhil D., Lakshmi D., Panchamoorthy Gopinath K., Arun J., Sivaramakrishnan R., Pugazhendhi A. (2021). A Critical Review on Production of Biopolymers from Algae Biomass and Their Applications. Bioresour. Technol..

[3] Bibire T., Yilmaz O., Ghiciuc C.M., Bibire N., Dănilă R. (2022). Biopolymers for Surgical Applications. Coatings.

[4] Varma K., Gopi S., Thomas S., Gopi S., Amalraj A. (2021). Chapter 7—Biopolymers and Their Role in Medicinal and Pharmaceutical Applications. Biopolymers and Their Industrial Applications.

[5] KloaregG B., Quatrano R.S. (1988). Structure of the Cell Walls of Marine Algae and Ecophysiological Functions of the Matrix Polysaccharides. Oceanogr. Mar. Biol..

[6] Bahsis L., Ablouh E.-H., Anane H., Taourirte M., Julve M., Stiriba S.-E. (2020). Cu( Ii )-Alginate-Based Superporous Hydrogel Catalyst for Click Chemistry Azide–Alkyne Cycloaddition Type Reactions in Water. RSC Adv..

[7] Stengel D.B., Connan S. (2015). Marine Algae: A Source of Biomass for Biotechnological Applications. Natural Products From Marine Algae.

[8] Mutanda T., Naidoo D., Bwapwa J.K., Anandraj A. (2020). Biotechnological Applications of Microalgal Oleaginous Compounds: Current Trends on Microalgal Bioprocessing of Products. Front. Energy Res..

[9] Li J., He J., Huang Y. (2017). Role of Alginate in Antibacterial Finishing of Textiles. Int. J. Biol. Macromol..

[10] Gheorghita Puscaselu R., Lobiuc A., Dimian M., Covasa M. (2020). Alginate: From Food Industry to Biomedical Applications and Management of Metabolic Disorders. Polymers.

[11] Cervino G., Fiorillo L., Herford A.S., Laino L., Troiano G., Amoroso G., Crimi S., Matarese M., D’Amico C., Nastro Siniscalchi E. (2018). Alginate Materials and Dental Impression Technique: A Current State of the Art and Application to Dental Practice. Mar. Drugs.

[12] Ablouh E.-H., Hanani Z., Eladlani N., Rhazi M., Taourirte M. (2019). Chitosan Microspheres/Sodium Alginate Hybrid Beads: An Efficient Green Adsorbent for Heavy Metals Removal from Aqueous Solutions. Sustain. Environ. Res..

[13] Ablouh E.-H., Essaghraoui A., Eladlani N., Rhazi M., Taourirte M. (2019). Uptake of Pb(II) onto Nanochitosan/Sodium Alginate Hybrid Beads: Mechanism and Kinetics Study. Water Environ. Res..

[14] Boussetta A., Ablouh E.-H., Benhamou A.A., Taourirte M., Moubarik A. (2021). Valorization of Moroccan Brown Seaweeds: Elaboration of Formaldehyde-Free Particleboards Based on Sodium Alginate–Corn-Starch—Mimosa Tannin Wood Adhesives. Int. J. Adhes. Adhes..

[15] Gopidas S.K., Subramani N. (2022). Recent Advances and Research Challenges in Bioprospecting Of Brown Seaweeds. Seaweed Biotechnology.

[16] Sangeetha J., Thangadurai D. (2022). Seaweed Biotechnology: Biodiversity and Biotechnology of Seaweeds and Their Applications.

[17] Rioux L.-E., Turgeon S.L., Tiwari B.K., Troy D.J. (2015). Chapter 7—Seaweed Carbohydrates. Seaweed Sustainability.

[18] Belattmania Z., Kaidi S., El Atouani S., Katif C., Bentiss F., Jama C., Reani A., Sabour B., Vasconcelos V. (2020). Isolation and FTIR-ATR and ^1^H NMR Characterization of Alginates from the Main Alginophyte Species of the Atlantic Coast of Morocco. Molecules.

[19] Draget K.I., Taylor C. (2011). Chemical, Physical and Biological Properties of Alginates and Their Biomedical Implications. Food Hydrocoll..

[20] Pawar S.N., Edgar K.J. (2012). Alginate Derivatization: A Review of Chemistry, Properties and Applications. Biomaterials.

[21] Chowdhury S., Chowdhury I.R., Kabir F., Mazumder M.A.J., Zahir M.H., Alhooshani K. (2019). Alginate-Based Biotechnology: A Review on the Arsenic Removal Technologies and Future Possibilities. J. Water Supply: Res. Technol.-Aqua.

[22] González R., Martín N., Seoane C., Marco J., Albert A., Cano F.H. (1992). The First Asymmetric Synthesis of Polyfunctionalized 4*H*-Pyrans via Michael Addition of Malononitrile to 2-Acyl Acrylates. Tetrahedron Lett..

[23] Shehab W.S., Ghoneim A.A. (2016). Synthesis and Biological Activities of Some Fused Pyran Derivatives. Arab. J. Chem..

[24] Saranya J., Benhiba F., Anusuya N., Subbiah R., Zarrouk A., Chitra S. (2020). Experimental and Computational Approaches on the Pyran Derivatives for Acid Corrosion. Colloids Surf. A Physicochem. Eng. Asp..

[25] Han H., Zhang Z.-F., Zhang J.-F., Zhang B. (2018). Pyran Derivatives: Anti-Breast Cancer Activity and Docking Study. Russ. J. Gen. Chem..

[26] Guo Z., Zhu W., Tian H. (2012). Dicyanomethylene-4*H*-Pyran Chromophores for OLED Emitters, Logic Gates and Optical Chemosensors. Chem. Commun..

[27] Xie Y., Wang Z., Wang D., Zhou Y., Lei Y., Gao W., Liu M., Huang X., Wu H. (2021). Reversible Photochromic Properties of 4,5,6-Triaryl-4*H*-Pyran Derivatives in a Solid State. Mater. Chem. Front..

[28] Tashrifi Z., Mohammadi-Khanaposhtani M., Hamedifar H., Larijani B., Ansari S., Mahdavi M. (2020). Synthesis and Pharmacological Properties of Polysubstituted 2-Amino-4*H*-Pyran-3-Carbonitrile Derivatives. Mol. Divers..

[29] Dekamin M.G., Peyman S.Z., Karimi Z., Javanshir S., Naimi-Jamal M.R., Barikani M. (2016). Sodium Alginate: An Efficient Biopolymeric Catalyst for Green Synthesis of 2-Amino-4*H*-Pyran Derivatives. Int. J. Biol. Macromol..

[30] Hecht H., Srebnik S. (2016). Structural Characterization of Sodium Alginate and Calcium Alginate. Biomacromolecules.

[31] Abka-khajouei R., Tounsi L., Shahabi N., Patel A.K., Abdelkafi S., Michaud P. (2022). Structures, Properties and Applications of Alginates. Mar. Drugs.

[32] Gorji S., Ghorbani-Vaghei R., Alavinia S. (2021). Sodium Alginate: Biopolymeric Catalyst for the Synthesis of 2-Amino-4-Arylthiazole Derivatives in Aqueous Medium. J. Mol. Struct..

[33] Pettignano A., Aguilera D.A., Tanchoux N., Bernardi L., Quignard F., Albonetti S., Perathoner S., Quadrelli E.A. (2019). Chapter 17—Alginate: A Versatile Biopolymer for Functional Advanced Materials for Catalysis. Studies in Surface Science and Catalysis.

[34] Kühbeck D., Mayr J., Häring M., Hofmann M., Quignard F., Díaz D.D. (2015). Evaluation of the Nitroaldol Reaction in the Presence of Metal Ion-Crosslinked Alginates. N. J. Chem..

[35] Zafari S., Ghorbani-Vaghei R., Alavinia S. (2022). Sodium Alginate: An Efficient Biopolymeric Catalyst for Green Synthesis of Phenylimidazo [1,2-a]Pyridine Derivatives. Polycycl. Aromat. Compd..

[36] Pathak T.S., Kim J.S., Lee S.-J., Baek D.-J., Paeng K.-J. (2008). Preparation of Alginic Acid and Metal Alginate from Algae and Their Comparative Study. J. Polym. Environ..

[37] Kaidi S., Belattmania Z., Bentiss F., Jama C., Reani A., Sabour B. (2021). Synthesis and Characterization of Silver Nanoparticles Using Alginate from the Brown Seaweed Laminaria Ochroleuca: Structural Features and Antibacterial Activity. Biointerface Res. Appl. Chem..

[38] Grasdalen H., Larsen B., Smidsrød O. (1979). A p.m.r. Study of the Composition and Sequence of Uronate Residues in Alginates. Carbohydr. Res..

[39] Draget K.I., Skjåk-Bræk G., Stokke B.T. (2006). Similarities and Differences between Alginic Acid Gels and Ionically Crosslinked Alginate Gels. Food Hydrocoll..

[40] Kolvari E., Koukabi N., Ozmaei Z., Khoshkho H., Seidi F. (2022). Synthesis of 2-Amino-4*H*-Pyran and 2-Benzylidene Malononitrile Derivatives Using a Basil Seed as a Cheap, Natural, and Biodegradable Catalyst. Curr. Res. Green Sustain. Chem..

[41] van der Helm M.P., Klemm B., Eelkema R. (2019). Organocatalysis in Aqueous Media. Nat. Rev. Chem..

[42] Banik B.K., Banerjee B. (2022). Organocatalysis: A Green Tool for Sustainable Developments.

[43] Saini S., Kaur N., Singh N. (2020). A Cytochrome C-Urea Functionalized Dipeptide Conjugate: An Efficient HBD Framework to Synthesize 4*H*-Pyrans via One-Pot Multicomponent Reaction. Green Chem..

[44] Hassanzadeh-Afruzi F., Dogari H., Esmailzadeh F., Maleki A. (2021). Magnetized Melamine-Modified Polyacrylonitrile (PAN@melamine/Fe3O4) Organometallic Nanomaterial: Preparation, Characterization, and Application as a Multifunctional Catalyst in the Synthesis of Bioactive Dihydropyrano [2,3-c]Pyrazole and 2-Amino-3-Cyano 4*H*-Pyran Derivatives. Appl. Organomet. Chem..

[45] Khazaei A., Abbasi F., Moosavi-Zare A.R. (2015). Catalytic Application of N,2-Dibromo-6-Chloro-3,4-Dihydro-2H-Benzo[e][1,2,4]Thiadiazine-7-Sulfonamide 1,1-Dioxide on the Synthesis of 1-Carbamato-Alkyl-2-Naphthols and 1-Thioamido-Alkyl-2-Naphthols. J. Sulfur Chem..

[46] Li J.P.H., Kennedy E.M., Adesina A.A., Stockenhuber M. (2019). Mechanistic Insights into the Knoevenagel Condensation Reaction over ZnO Catalysts: Direct Observation of Surface Intermediates Using in Situ FTIR. J. Catal..

[47] El Jemli Y., Khallouk K., Lanaya S., Brulé M., Barakat A., Abdelouahdi K., Solhy A. (2022). Hybrid Alginate–Brushite Beads Easily Catalyze the Knoevenagel Condensation On-Water. ACS Omega.

[48] Pagadala R., Maddila S., Jonnalagadda S.B. (2015). An Efficient, Multicomponent, One-pot Synthesis of Tetra Substituted Pyrans in Water. J. Heterocycl. Chem..

[49] Ardiles C.S., Rodríguez C.C. (2021). Theoretical Study for Determining the Type of Interactions between a GG Block of an Alginate Chain with Metals Cu^2+^, Mn^2+^, Ca^2+^ and Mg^2+^. Arab. J. Chem..

[50] Costa M.P.M., Prates L.M., Baptista L., Cruz M.T.M., Ferreira I.L.M. (2018). Interaction of Polyelectrolyte Complex between Sodium Alginate and Chitosan Dimers with a Single Glyphosate Molecule: A DFT and NBO Study. Carbohydr. Polym..

[51] Plazinski W., Plazinska A. (2011). Molecular Dynamics Study of the Interactions between Phenolic Compounds and Alginate/Alginic Acid Chains. N. J. Chem..

[52] Knani D., Barkay-Olami H., Alperstein D., Zilberman M. (2017). Simulation of Novel Soy Protein-Based Systems for Tissue Regeneration Applications. Polym. Adv. Technol..

[53] Kalla R.M.N., Kim M.R., Kim I. (2015). Dibutylamine-Catalysed Efficient One-Pot Synthesis of Biologically Potent Pyrans. Tetrahedron Lett..

[54] Amirnejat S., Nosrati A., Peymanfar R., Javanshir S. (2020). Synthesis and Antibacterial Study of 2-Amino-4*H*-Pyrans and Pyrans Annulated Heterocycles Catalyzed by Sulfated Polysaccharide-Coated BaFe12O19 Nanoparticles. Res. Chem. Intermed..

[55] Hakiminasab S., Habibi A., Shahcheragh S.M., Farahani Y., Sardari S., Dolati H., Mahdavi S.M., Habibi M. (2021). Efficient Pyran Derivatives Synthesis in DES Medium and Their Antimicrobial Evaluation as Inhibitors of Mycobacterium Bovis (BCG). J. Iran. Chem. Soc..

[56] Kharbangar I., Rohman M.R., Mecadon H., Myrboh B. (2012). KF-Al_2_O_3_ as an Efficient and Recyclable Basic Catalyst for the Synthesis of 4*H*-Pyran-3-Carboxylates and 5-Acetyl-4*H*-Pyrans. Int. J. Org. Chem..

[57] Ahmad I., Jasim S.A., Yasin G., Al-Qargholi B., Hammid A.T. (2022). Synthesis and Characterization of New 1,4-Dihydropyran Derivatives by Novel Ta-MOF Nanostructures as Reusable Nanocatalyst with Antimicrobial Activity. Front. Chem..

[58] Frisch M.J., Trucks G.W., Schlegel H.B., Scuseria G.E., Robb M.A., Cheeseman J.R., Scalmani G., Barone V., Mennucci B., Petersson G.A. (2009). Gaussian 09.

[59] Becke A.D. (1993). Density-functional Thermochemistry. III. The Role of Exact Exchange. J. Chem. Phys..

[60] Lee C., Yang W., Parr R.G. (1988). Development of the Colle-Salvetti Correlation-Energy Formula into a Functional of the Electron Density. Phys. Rev. B.

[61] Takano Y., Houk K.N. (2005). Benchmarking the Conductor-like Polarizable Continuum Model (CPCM) for Aqueous Solvation Free Energies of Neutral and Ionic Organic Molecules. J. Chem. Theory Comput..

[62] Lu T., Chen F. (2012). Multiwfn: A Multifunctional Wavefunction Analyzer. J. Comput. Chem..

[63] Humphrey W., Dalke A., Schulten K. (1996). VMD: Visual Molecular Dynamics. J. Mol. Graph..

[64] Johnson E.R., Keinan S., Mori-Sánchez P., Contreras-García J., Cohen A.J., Yang W. (2010). Revealing Noncovalent Interactions. J. Am. Chem. Soc..

