# Supplementary material for: Mechanistic Insights into the Selective Synthesis of 4H-Pyran Derivatives On-Water Using Naturally Occurring Alginate from Sargassum muticum: Experimental and DFT Study
Source: Gels. 2022 Nov 4;8(11):713. doi: 10.3390/gels8110713 (PMC9689221; doi:10.3390/gels8110713)
Supplement: Supplementary file 1 [file gels-08-00713-s001.zip › gels-1953336-supplementary.pdf]

gels

Article

Mechanistic Insights into the Selective Synthesis of 4*H*-Pyran Derivatives On-Water Using Naturally Occurring Alginate from *Sargassum muticum:* Experimental and DFT Study

Khaoula Oudghiri [1], Zahira Belattmania [2,*], Hamid Elmouli [3], Salaheddine Guesmi [3], Fouad Bentiss [4,5], Brahim Sabour [2], Lahoucine Bahsis [3,6,*] and Moha Taourirte [1]

1 Laboratoire de Recherche en Développement Durable et Santé, Faculté des Sciences et Techniques de Marrakech, Université Cadi Ayyad, Marrakech 40000, Morocco
2 Laboratory of Plant Biotechnology, Ecology and Ecosystem Valorization—URL CNRST N=10, Faculty of Sciences El Jadida, University Chouaib Doukkali, P.O. Box 20, El Jadida 24000, Morocco
3 Laboratoire de Chimie de Coordination et d'Analytique (LCCA), Département de Chimie, Faculté des Sciences, Université Chouaïb Doukkali, P.O. Box 20; El Jadida 24000, Morocco
4 Laboratory of Catalysis and Corrosion of Materials, Faculty of Sciences, University Chouaib Doukkali, P.O. Box 20, El Jadida 24000, Morocco
5 Materials and Transformations Unit, University of Lille, CNRS, INRAE, Centrale Lille, UMR 8207-UMET, F-59000 Lille, France
6 Laboratoire de Chimie Analytique et Moléculaire, LCAM, Faculté Polydisciplinaire de Safi, Université Cadi Ayyad, Safi 46030, Morocco
* Correspondence: belattmania.z@ucd.ac.ma (Z.B.); bahsis.lahoucine@gmail.com (L. B.)

Supplementary Materials

1. SEM/EDX analyses of fresh and recovered SA

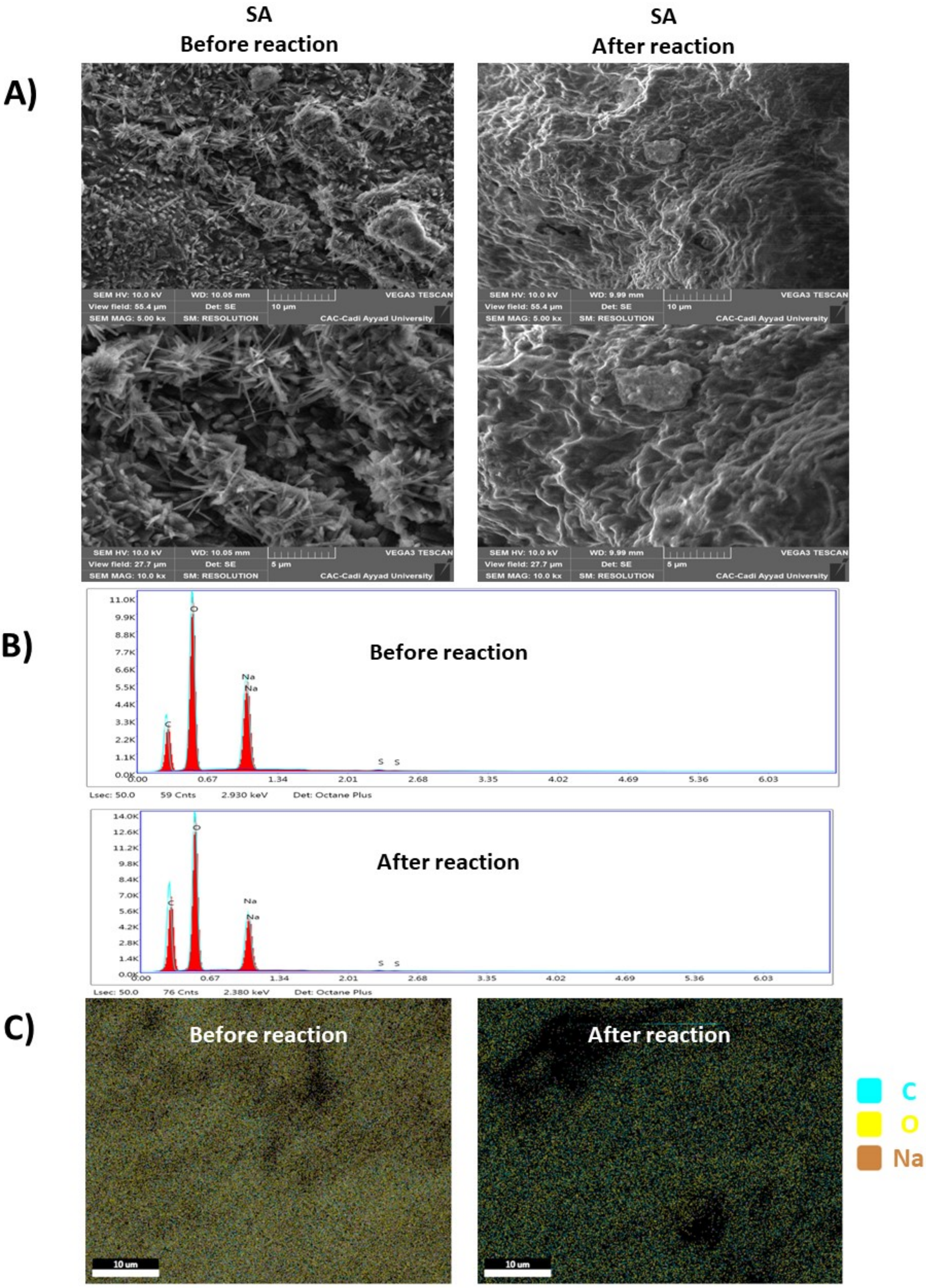

Figure S1. (**A**) SEM micrographs, (**B**) EDX and (**C**) EDX mapping analyses of fresh and recovered natural-occurring SA surface.

2. NMR analysis of prepared compound

2.1. Methyl 6-amino-5-cyano-2-methyl-4-phenyl-4H-pyran-3-carboxylate (4a)

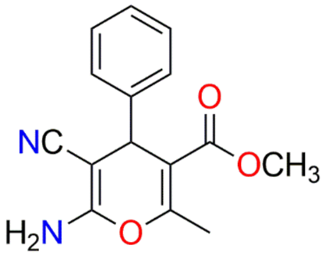

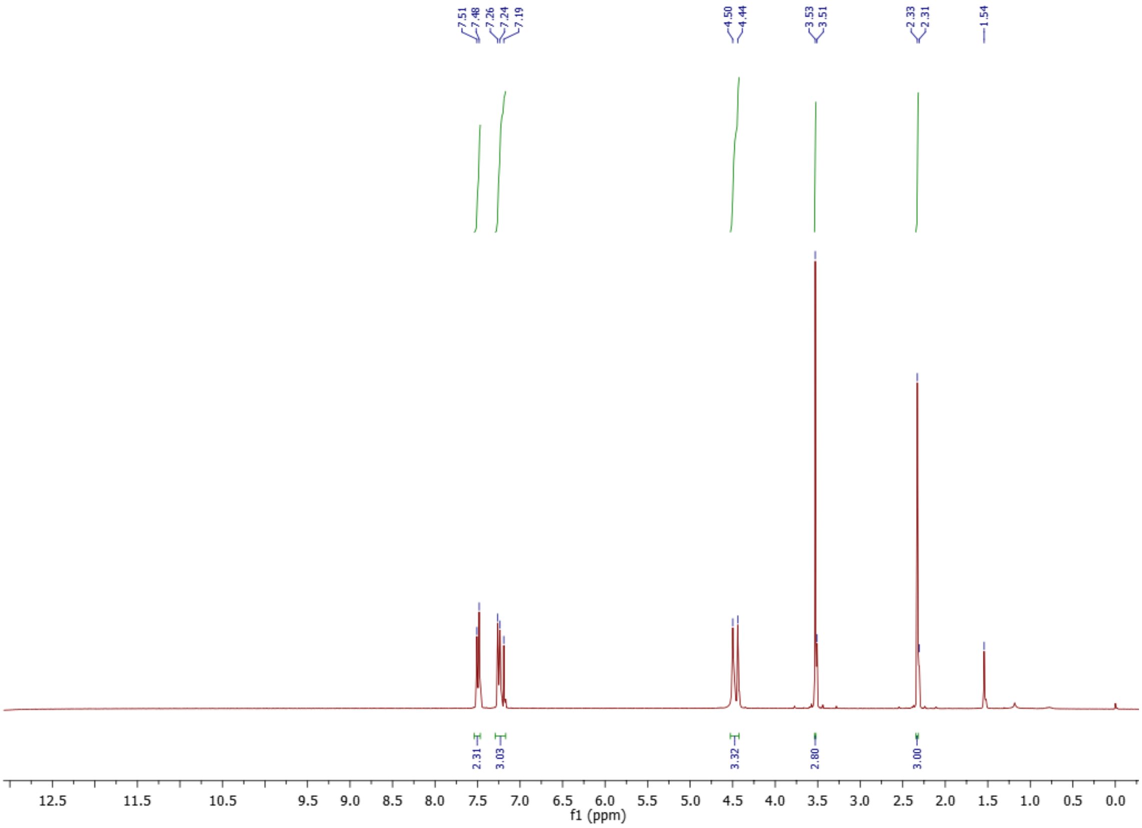

Figure S2. 1H NMR Spectrum of compound 4a.

Yield: 93.7 %, white crystals, mp 172-174 °C, 1H NMR (300 MHz, CDCl3) δ 7.49 (d, J = 8.1 Hz, 5H), 7.25 (d, J = 8.1 Hz, 5H), 4.50 (s, 2H), 4.44 (s, 1H), 3.52 (d, J = 6.4 Hz, 3H), 2.33 (s, 3H).

2.2. Methyl 6-amino-4-(2-bromophenyl)-5-cyano-2-methyl-4H-pyran-3-carboxylate (4b)

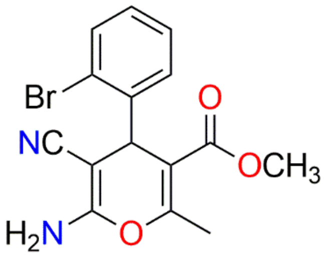

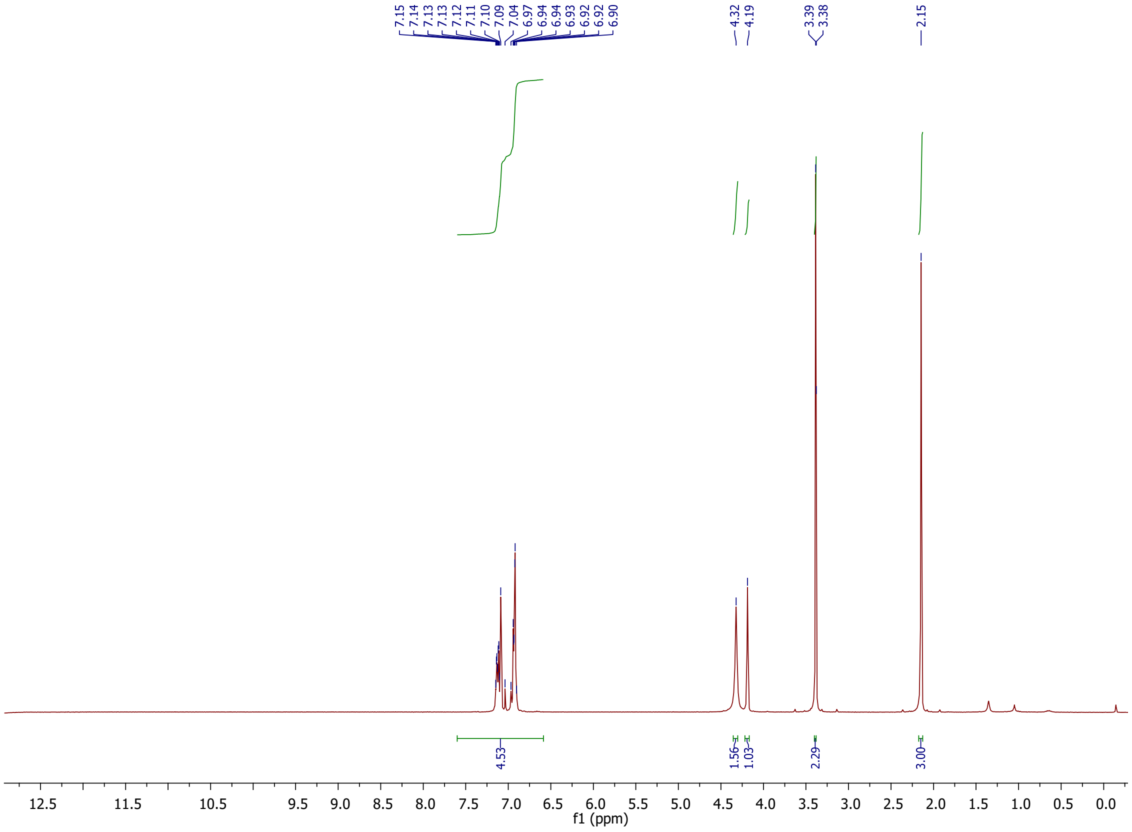

Figure S3. 1H NMR Spectrum of compound 4b.

Yield: 50,2%, white crystals, mp 172-174 °C, 1H NMR (300 MHz, CDCl3) δ 7.60 – 6.59 (m, 1H), 4.32 (s, 2H), 4.19 (s, 1H), 3.39 (s, 3H), 2.15 (s, 3H).

2.3. Methyl 6-amino-5-cyano-4-(3-fluorophenyl)-2-methyl-4H-pyran-3-carboxylate (4c)

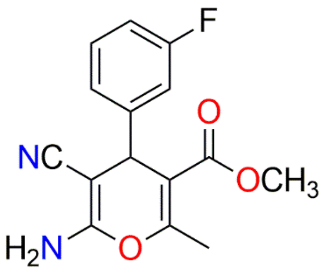

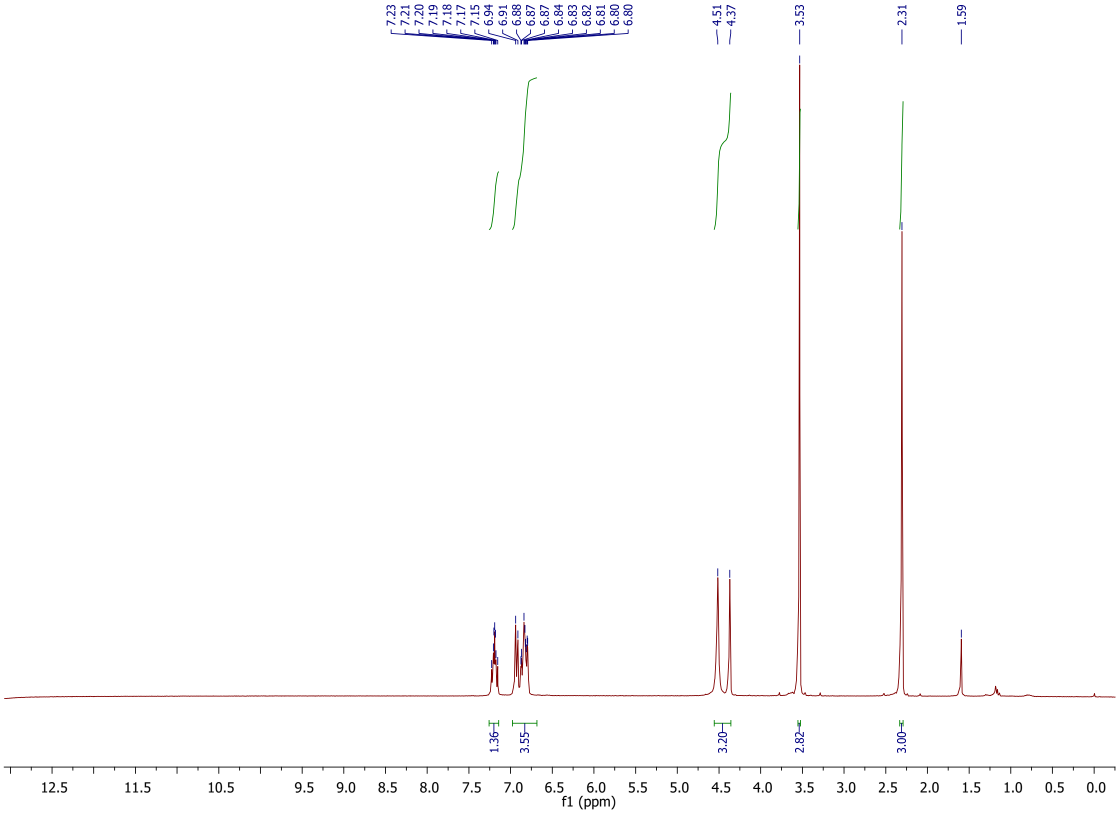

Figure S4. 1H NMR Spectrum of compound 4c.

Yield: 77,3%, white crystals, mp 170-172 °C, 1H NMR (300 MHz, CDCl3) δ 7.26 – 7.14 (m, 1H), 6.98 – 6.68 (m, 2H), 4.44 (s, J = 43.2 Hz, 2H), 3.53 (s, 3H), 2.31 (s, 3H).

2.4. Methyl 6-amino-5-cyano-2-methyl-4-(4-nitrophenyl)-4H-pyran-3-carboxylate (4d)

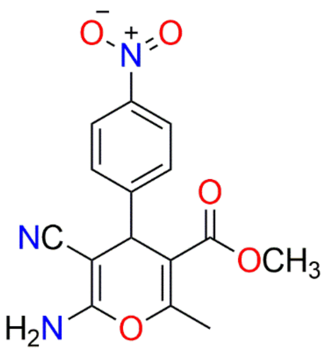

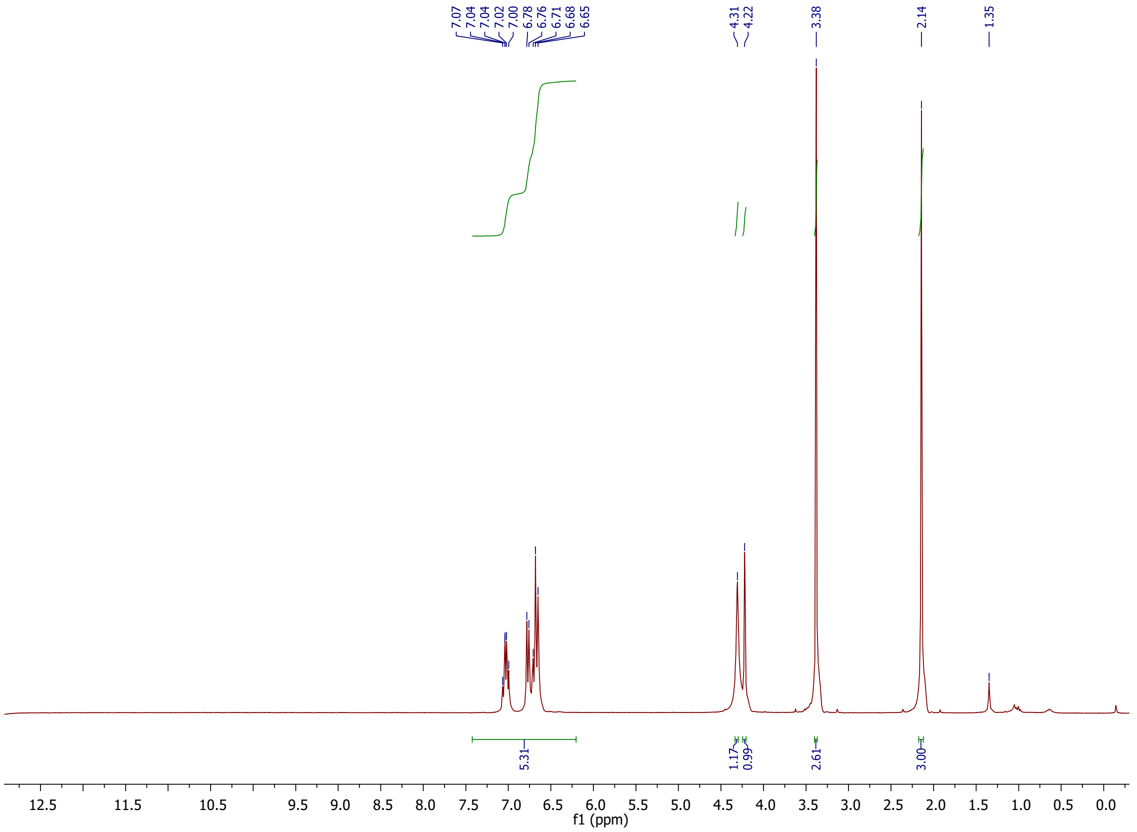

Figure S5. 1H NMR Spectrum of compound 4d.

Yield: 52,1%, white solid, mp 180-181 °C, 1H NMR (300 MHz, CDCl3) δ 7.42 – 6.20 (m, 1H), 4.31 (s, 2H), 4.22 (s, 1H), 3.38 (s, 3H), 2.14 (s, 3H).

2.5. Methyl 6-amino-5-cyano-2-methyl-4-(4-(trifluoromethyl)phenyl)-4H-pyran-3-carboxylate (4e)

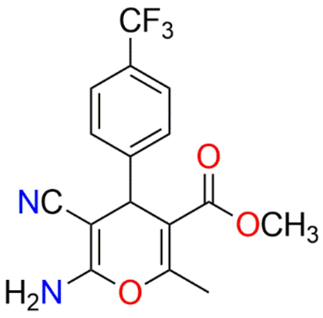

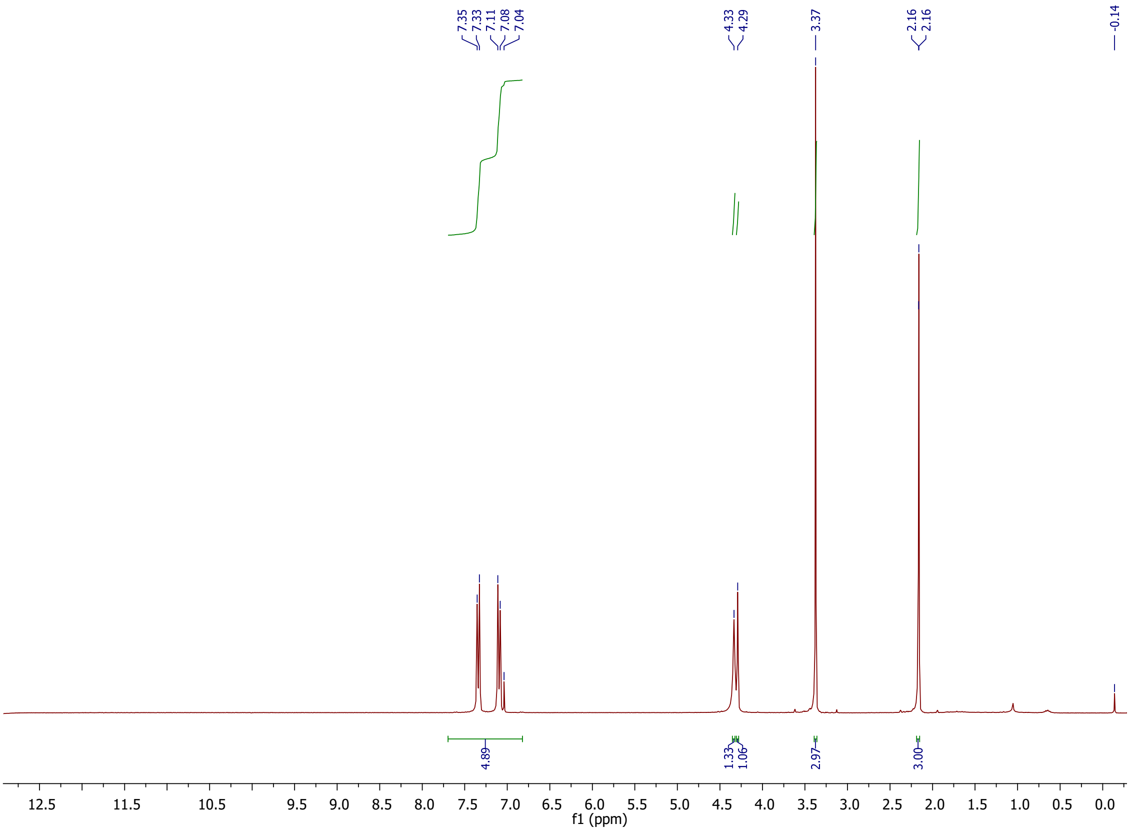

Figure S6. 1H NMR Spectrum of compound 4e.

Yield: 82,3%, white solid, mp 173-175 °C, 1H NMR (300 MHz, CDCl3) δ 7.34 (d, J = 8.2 Hz, 1H), 7.10 (d, J = 8.1 Hz, 1H), 7.04 (s, 1H), 4.33 (s,2), 4.29 (s, 1H), 3.37 (s, 3H), 2.16 (s, J = 0.8 Hz, 3H).

2.6. Methyl 6-amino-5-cyano-2-methyl-4-(4-(methylphenyl)-4H-pyran-3-carboxylate (4g)

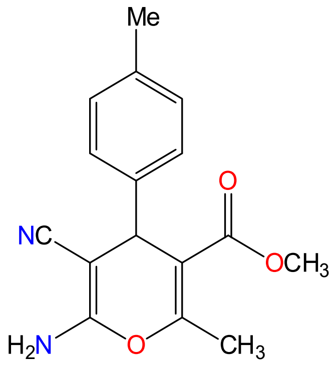

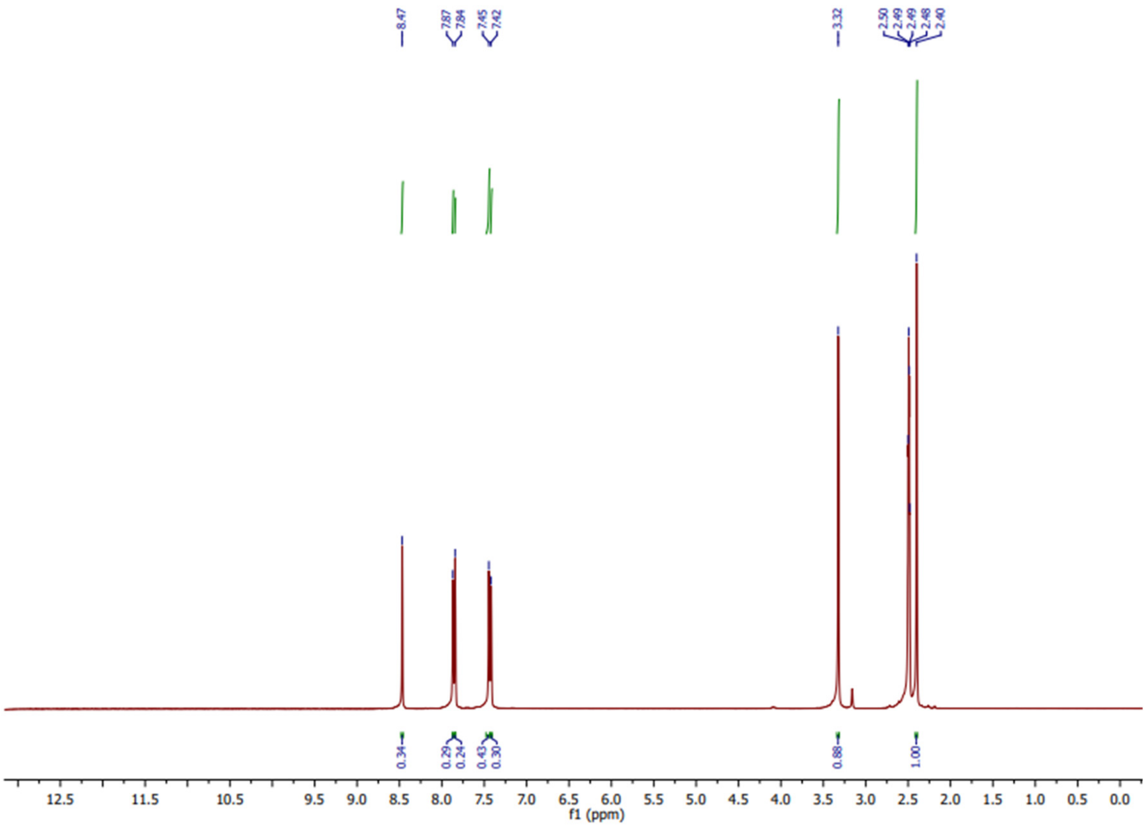

Figure S7. 1H NMR Spectrum of compound 4g.

Yield: 82%, white crystals, mp 143-146°C, 1H NMR (300 MHz, DMSO) δ 7.42-7.80 (m, J = 8.2 Hz, 1H), 3.32 (s, 3H), 2.40 (s, J = 0.8 Hz, 3H),8.47(s,3H).

2.7. Methyl 6-amino-5-cyano-2-methyl-4-(4-(chlorophenyl)-4H-pyran-3-carboxylate (4f)

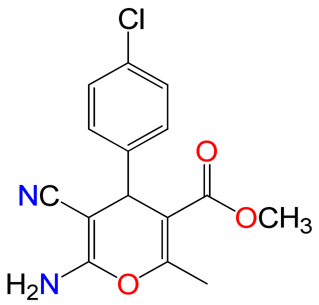

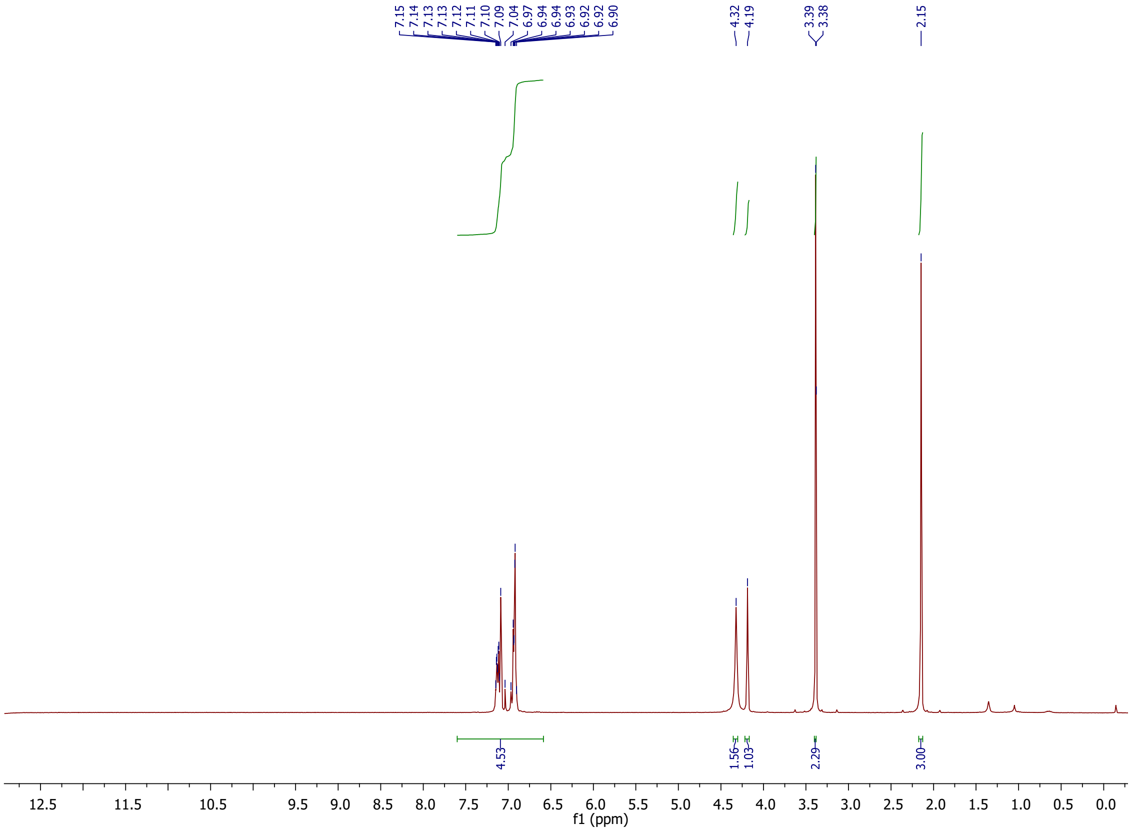

Figure S8. 1H NMR Spectrum of compound 4f.

Yield: 82%, white solid, mp 160-163°C, 1H NMR (300 MHz, DMSO) δ 7.34 (d, J = 8.2 Hz, 1H), 7.20 (d, J = 8.1 Hz, 1H), 7.07 (s, 1H), 4.46 (s,2H), 4.37 (s, 1H), 3.53 (s, 3H), 2.30 (s, J = 0.8 Hz, 3H).

2.8. Methyl 6-amino-5-cyano-2-methyl-4-(4-(dimethylaminophenyl)-4H-pyran-3-carboxylate (4h)

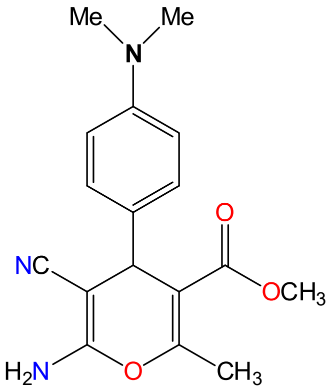

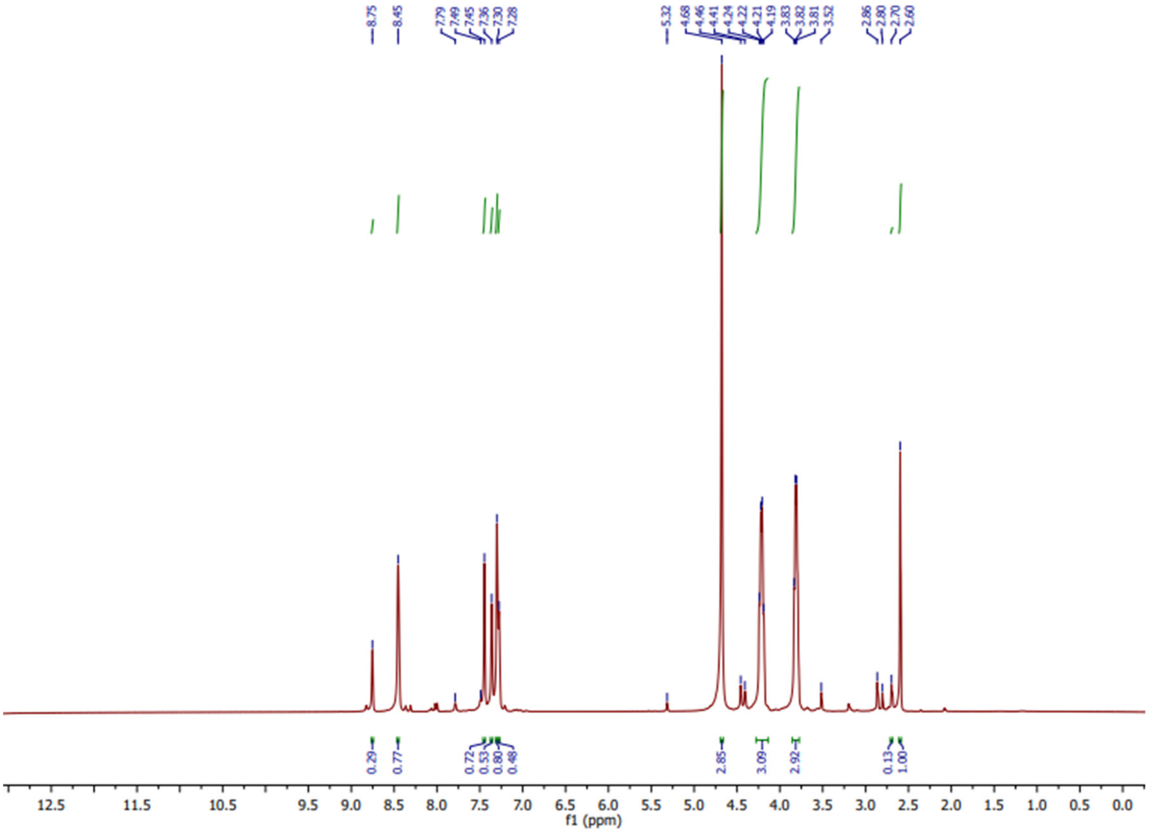

Figure S9. 1H NMR Spectrum of compound 4h.

Yield: 95%, white solid, mp 195-197°C, 1H NMR (300 MHz, DMSO) δ 8.75 (s, 1H), 8.45 (s, 1H), 7.28-7.49 (M, J = 8.2 Hz, 1H), 4.68 (s,2H), 4.19 (s, 1H),3.81 (s,3H), 2.58 (s, J = 0.8 Hz, 3H).

3. FTIR spectrum of benzaldehyde

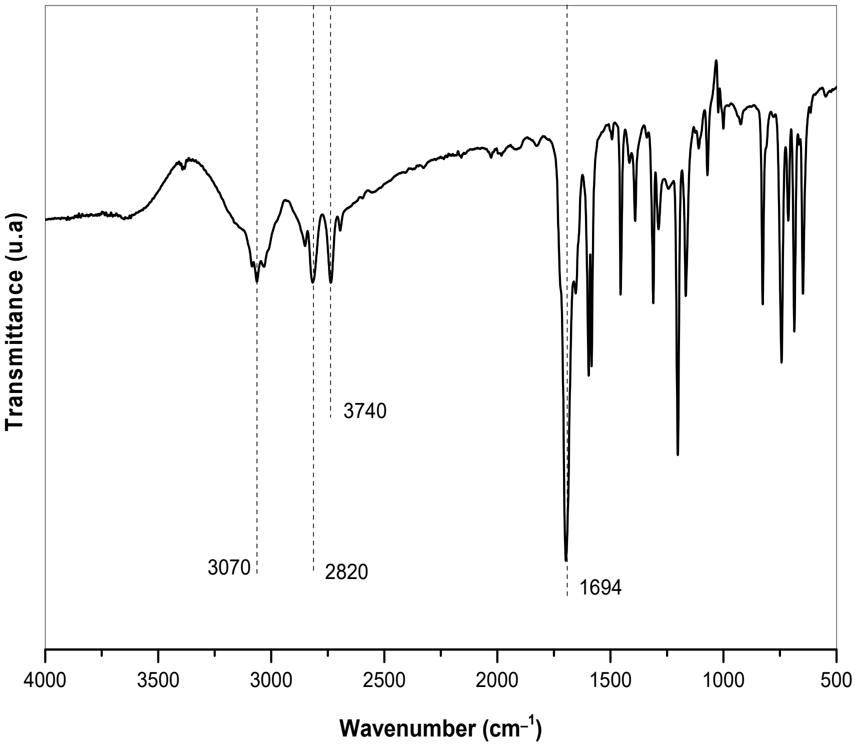

Figure S10. FTIR spectrum of benzaldehyde.

4. NCI analysis of RC intermediate

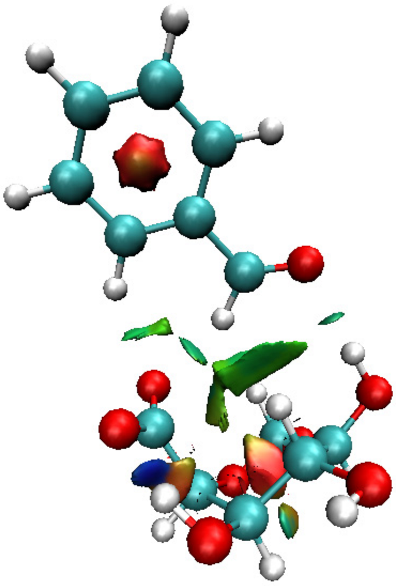

Figure S11. NCI analysis of RC intermediate (s = 0.34). For color codes: green (carbon atoms), red (oxygen atoms), blue (nitrogen atoms) and white (hydrogen atoms).

5. Effect of the amount of SA on the pH of the aqueous solution

Table S1. Effect of the amount of SA on the pH of aqueous solution[a].

Amount of SA (mg)	pH
5	8.07
20	9.83
40	10.02
80	10.30
100	10.34

[a]Reaction condition: water (5 mL) at room temperature.

6. DFT calculation of non-catalytic reaction for selective synthesis of the product 4a

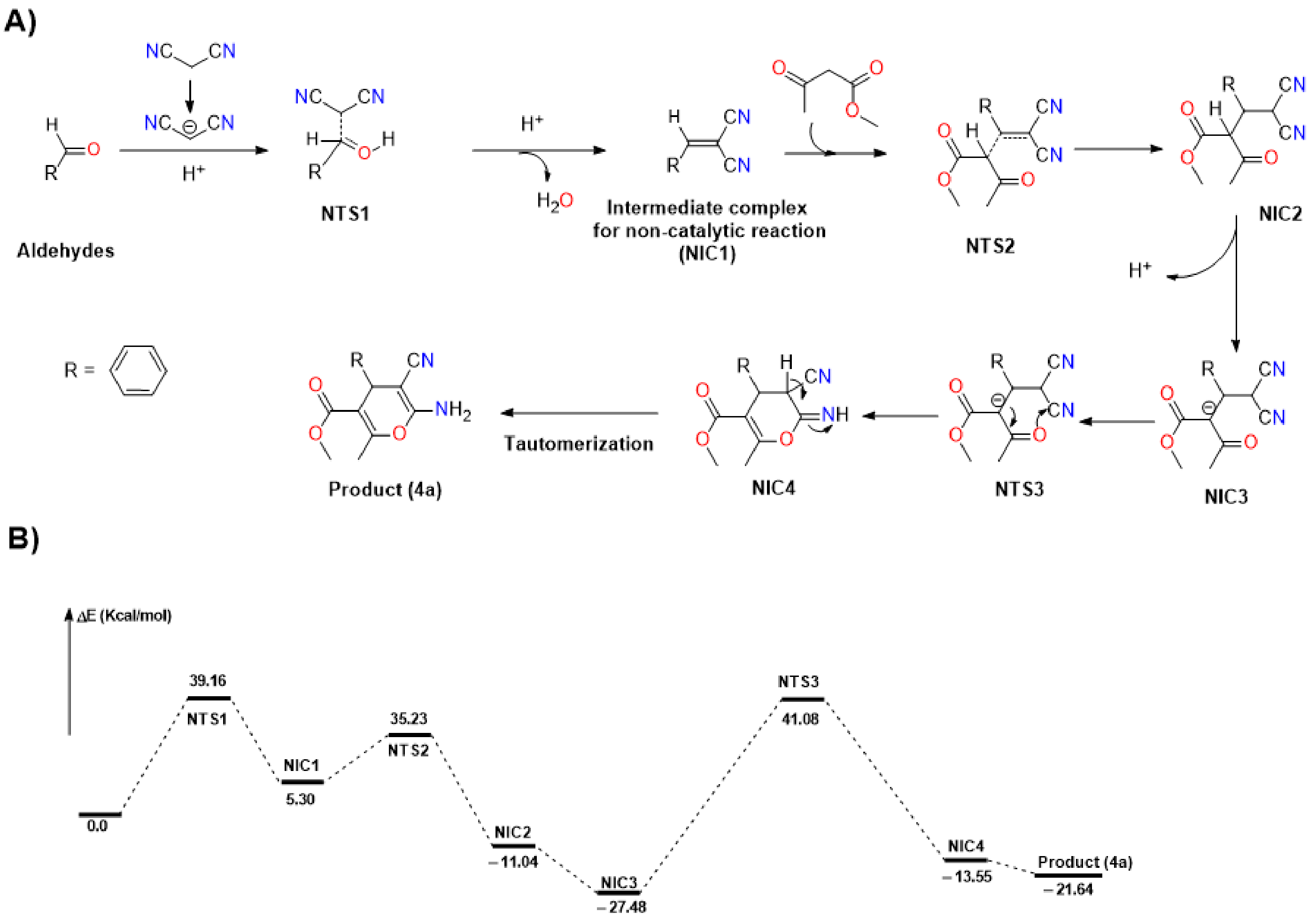

Figure S12. (**A**) Generally accepted mechanism and (**B**) DFT-computed energy profile for the non-catalytic reaction for the synthesis of 4a in water. All values are reported in kcal/mol.

7. Characterization of the recovered SA

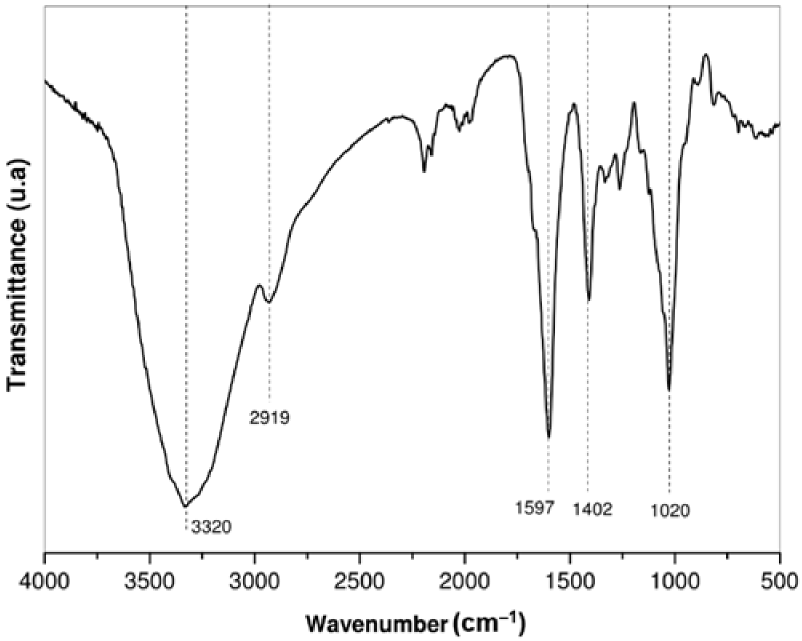

Figure S13. FTIR spectrum of the recovered SA after two cycles.
